# Supplementary material for: Structural dynamics of RAF1-HSP90-CDC37 and HSP90 complexes reveal asymmetric client interactions and key structural elements
Source: Commun Biol. 2024 Mar 2;7:260. doi: 10.1038/s42003-024-05959-3 (PMC10908828; doi:10.1038/s42003-024-05959-3)
Supplement: Supplementary file 2 — Supplementary Materials [file 42003_2024_5959_MOESM2_ESM.pdf]

Structural dynamics of RAF1-HSP90-CDC37 and HSP90 complexes reveal asymmetric client interactions and key structural elements

Supplementary Information

- Supplementary Figure 1-22
- Supplementary Table 1
- Supplementary Methods

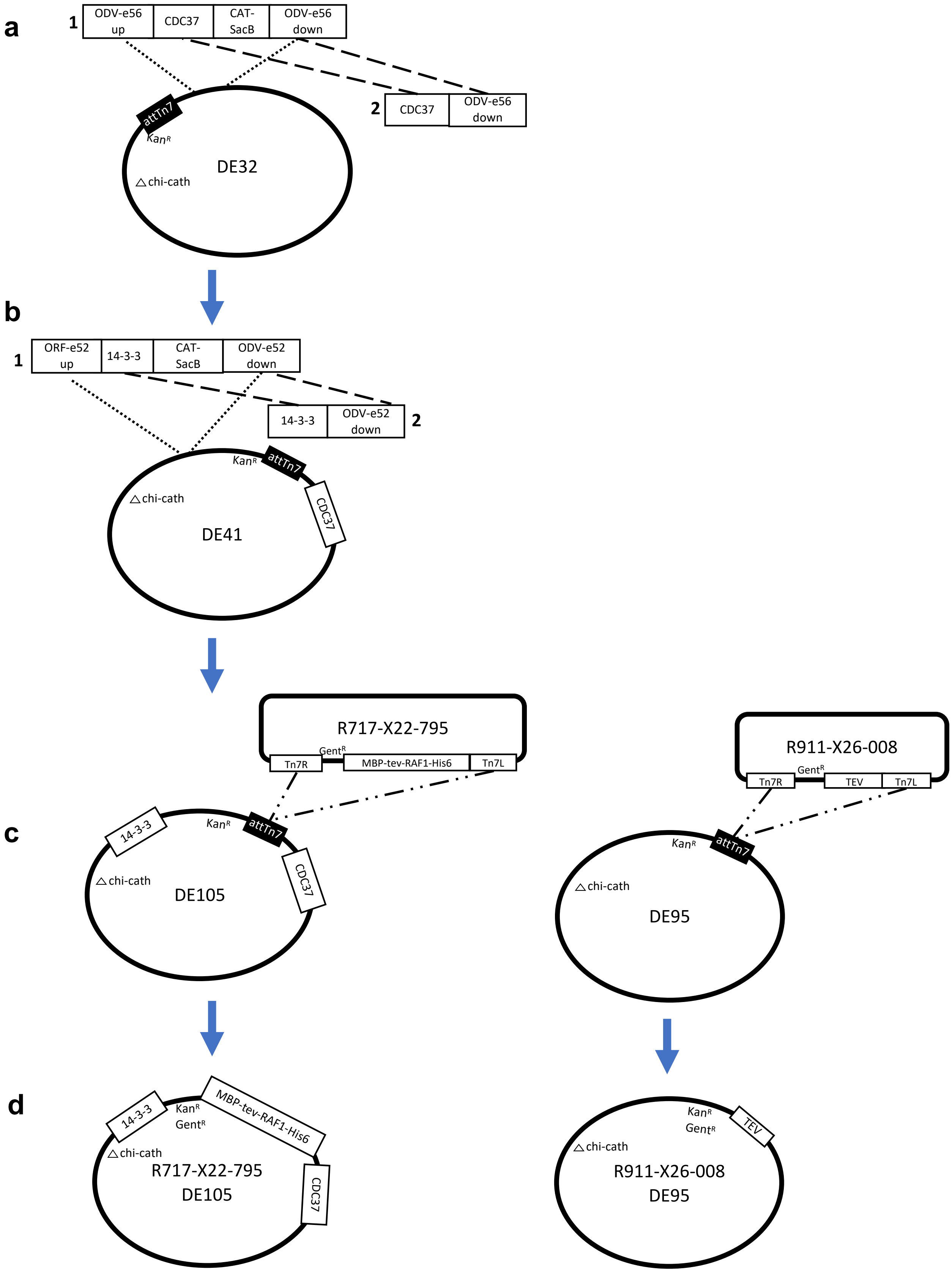

Supplementary Figure 1. Construction of bacmids for infection and co-expression in insect cells. **a.** Addition of CDC37 to bmon14272 delta *chi-cath* (DE32) to generate DE41. 1- Knock-in cassette for CDC37, 2-Marker removal cassette. **b.** Addition of 14-3-3 to bmon14272 delta *chi-cath*, ORF-52:CDC37 (DE41) to generate DE105. 1-Knock-in cassette for 14-3-3, 2-Marker removal cassette. **c.** Bac-to-Bac transposition with expression plasmids DE105 and DE95. **d.** Final bacmid DNAs used to generate baculoviruses R717-X22-795-DE105 and R911-X26-008-DE95.

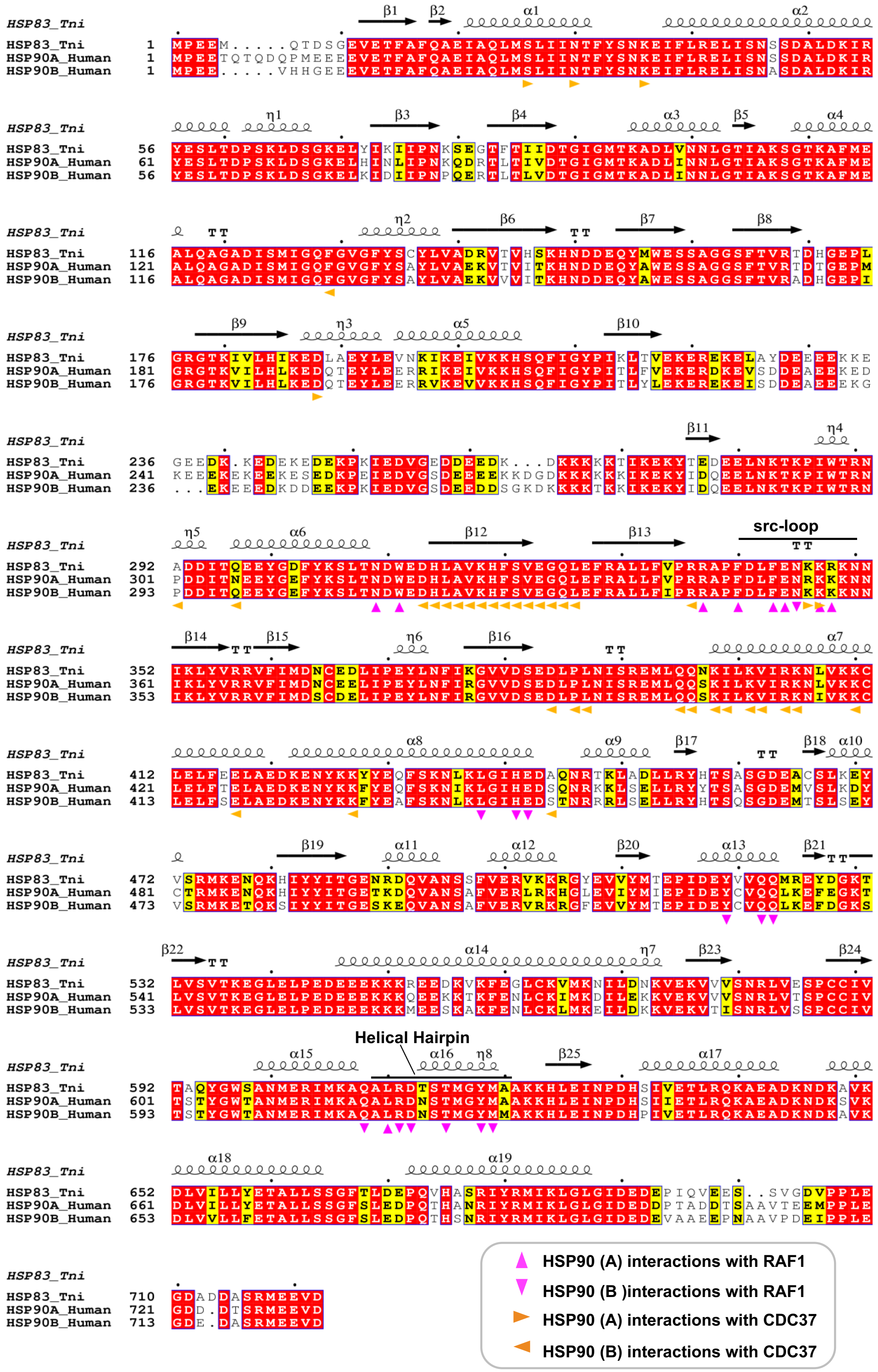

Supplementary Figure 2. Amino acid sequence alignment of insect and human HSP proteins. The multiple sequence alignment shows HSP protein from *Trichoplusia ni* aligned with human HSP90 alpha and beta proteins. The alignment was performed using ClustalW (*Nucleic Acids Res.*, **22**(22), 4673–4680), and the image was generated using ESPript 3.0 (*Nucleic Acids Res.*, **42**(W1), W320-W324). On top of the sequence alignment, the secondary structure and the structural elements that form the luminal cavity are depicted, including the src-loop (MD) and helical hairpin (CTD) of HSP90. The interactions formed by HSP residues with RAF1 and CDC37 are also mapped onto the sequence alignment.

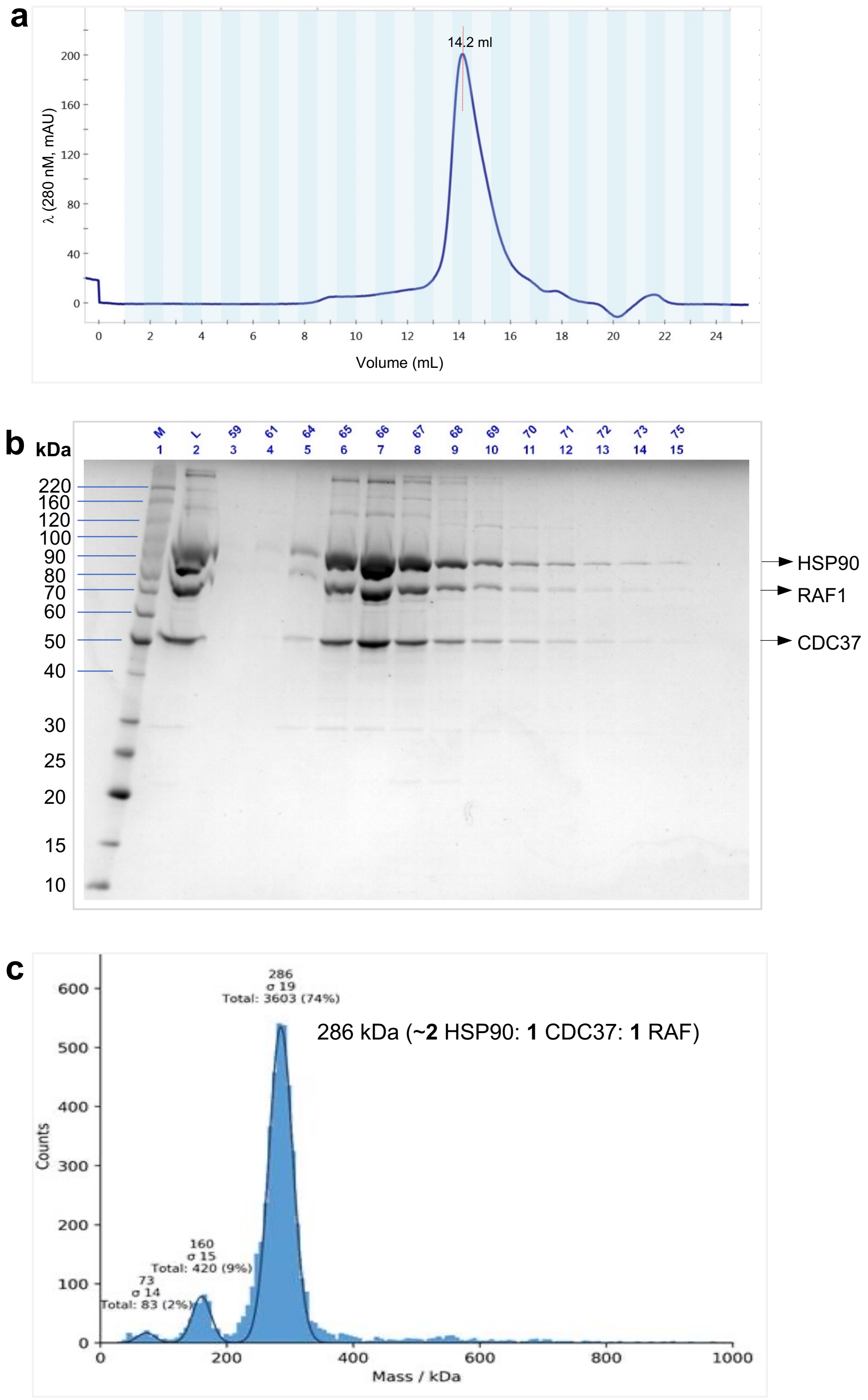

Supplementary Figure 3. Purification of RAF1-HSP90-CDC37 complex. a. Elution profile from the size-exclusion column chromatography showing peak fractions corresponding to the RAF1-HSP90-CDC37 complex. **b.** SDS-PAGE analysis of the peak fractions showing bands associated with HSP90, RAF1, and CDC37. Marker and load are in lanes 1 and 2, whereas peak fractions from 59 to 75 corresponding to the peak in panel a are in lanes 3-15 **c.** Mass photometry analysis of the purified RAF1-HSP90-CDC37 complex. Data were fit to Gaussian distributions, with mean molecular mass indicated above the peak.

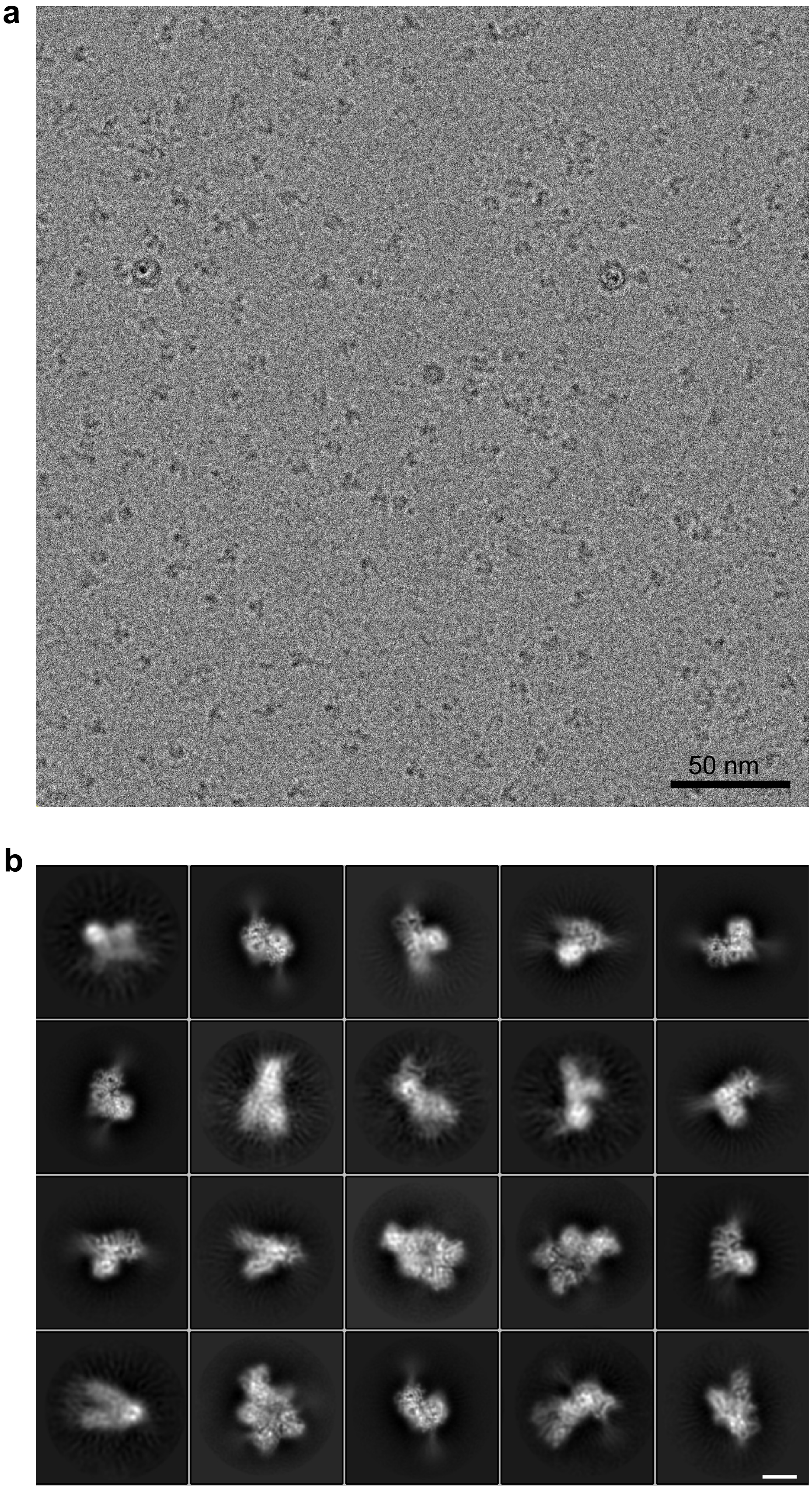

Supplementary Figure 4. Cryo-EM analysis of the purified RAF1-HSP90-CDC37 complex. a. Exemplar electron micrograph of a cryogenically preserved sample of the RAF1-HSP90-CDC37 complex applied to grids. The scale bar represents 50 nm. **b.** Exemplar 2D classification of particles picked from the micrograph. The scale bar represents 50 Å.

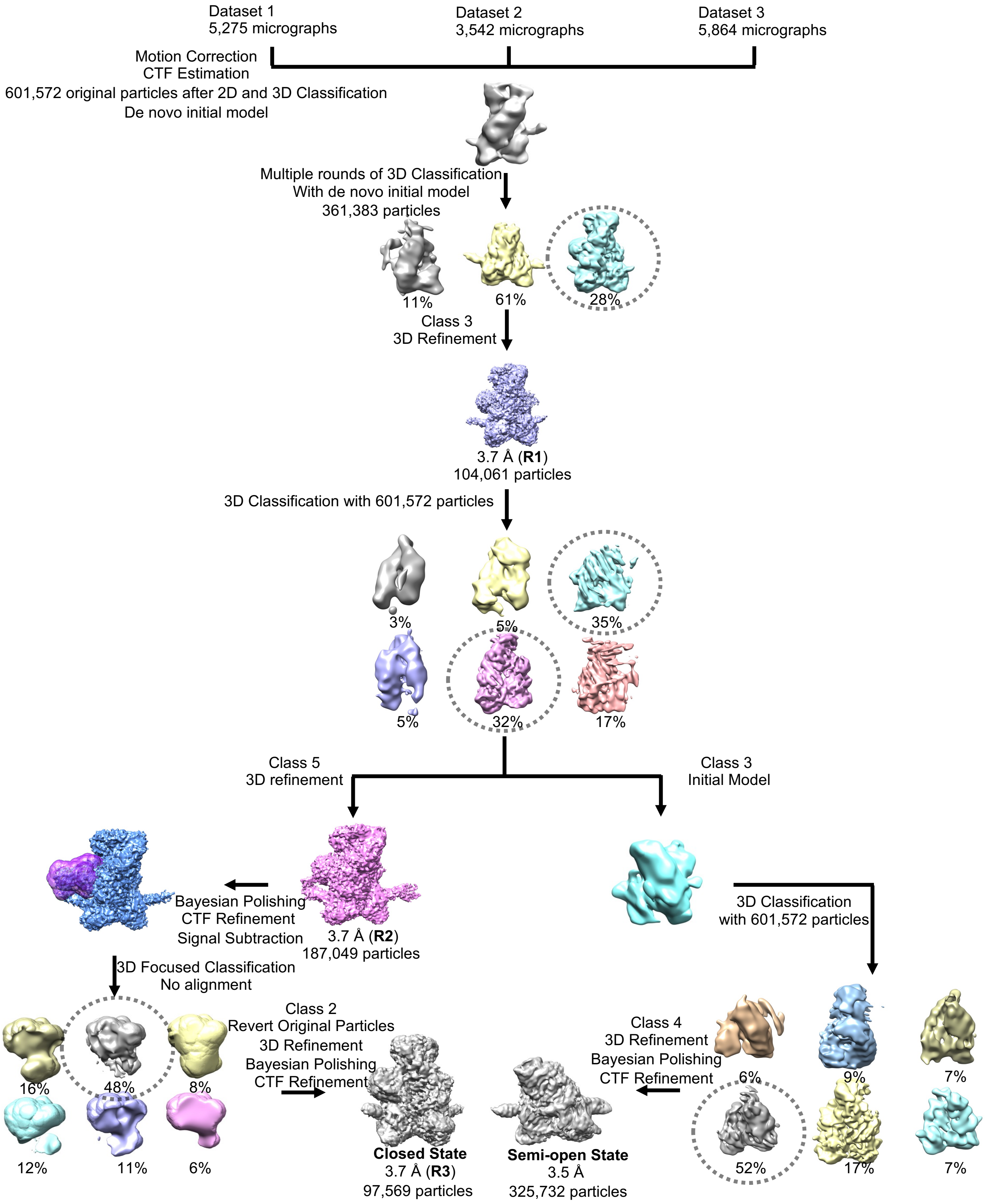

Supplementary Figure 5. Cryo-EM processing workflow. A schematic representation of the data processing workflow in RELION. For details, see the Materials and Methods section of the Image Processing section. Representative cryo-EM maps and masks are depicted at various stages of image processing that yielded both the closed and semi-open states. The number of particles and the global resolution of some of the classes are indicated. The three different reconstructions for the closed state are designated R1, R2, and R3.

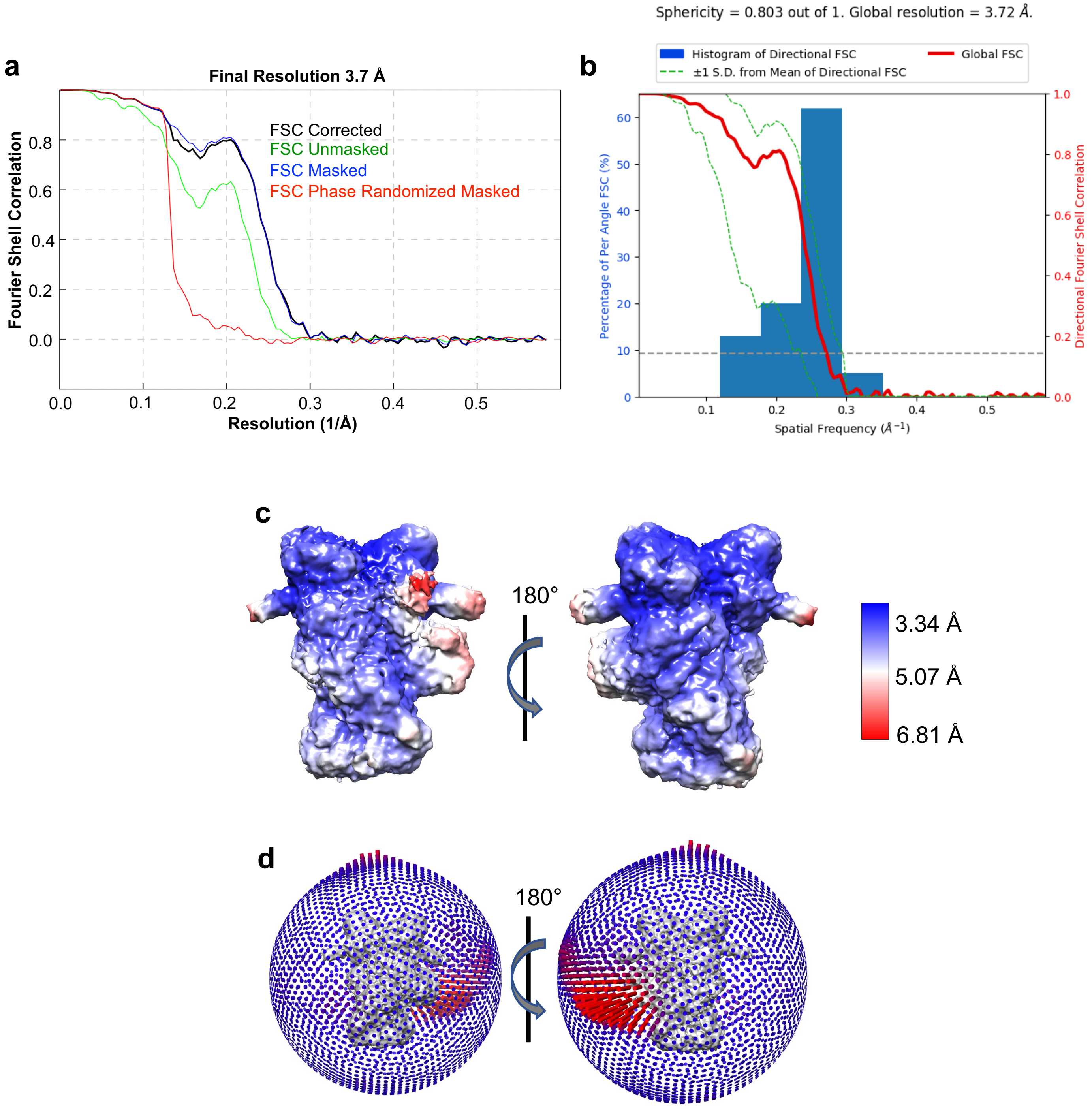

Supplementary Figure 6. Characteristics of the map associated with the RAF1-HSP90-CDC37 complex in the closed state. a. The gold-standard Fourier Shell Correlation (FSC) curve for the 3D reconstruction of the closed state. **b.** Directional FSC histograms and 3DFSC curves. **c.** Surface representation of the RAF1-HSP90-CDC37 complex, colored to reflect the local resolution calculated by Phenix Local Resolution. **d.** The angular distribution of the final reconstruction. Each column represents one view, and the size of the column is proportional to the number of particles in that view.

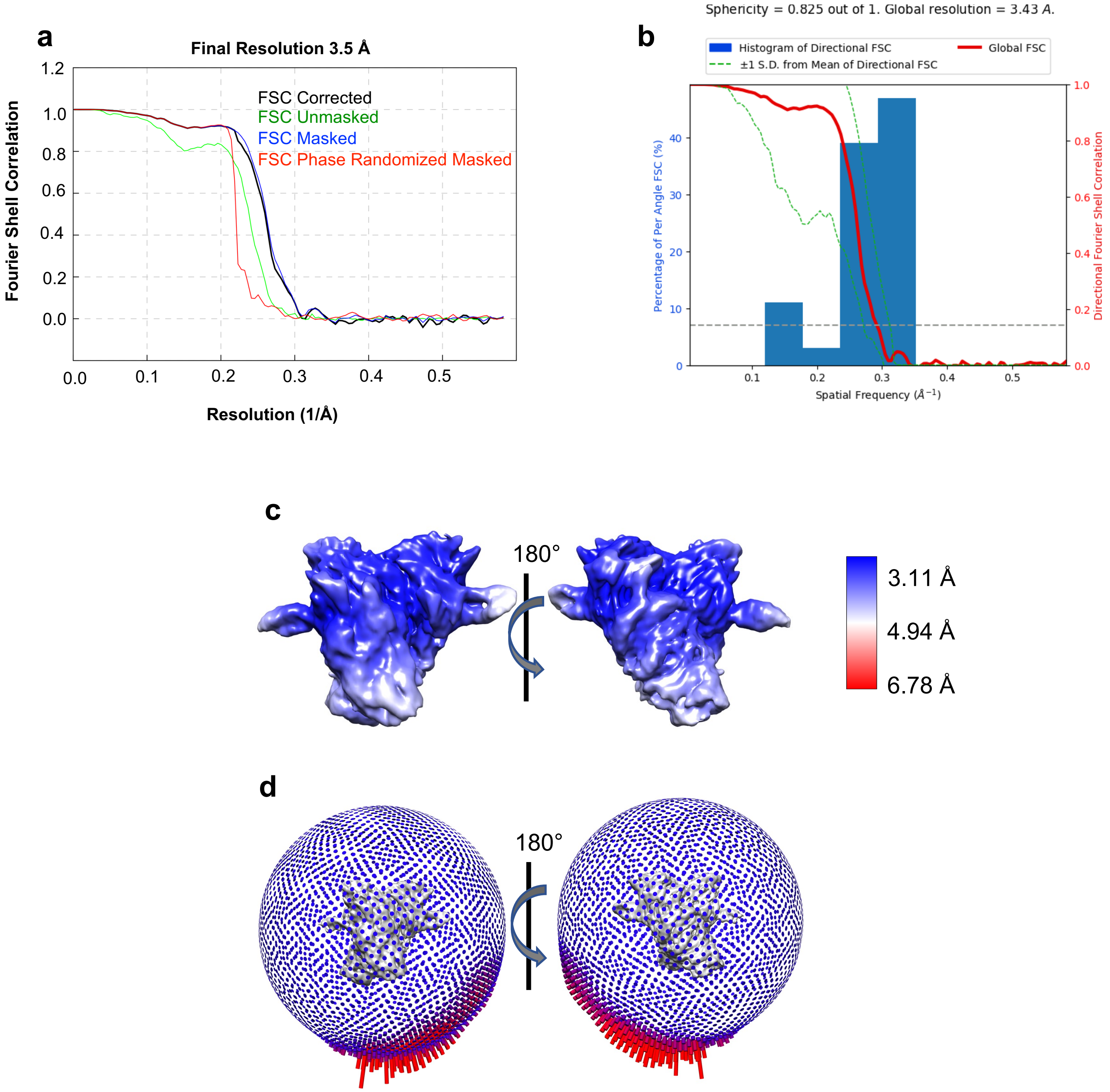

Supplementary Figure 7. Characteristics of the map associated with the HSP90 dimeric complex in the semi-open state. a. The gold-standard Fourier Shell Correlation (FSC) curve for the 3D reconstruction of the semi-open state. **b.** Directional FSC histograms and 3DFSC curves are shown in this panel. **c.** Surface representation of the dimeric HSP90 complex, colored to reflect the local resolution calculated by Phenix Local Resolution. **d.** The angular distribution of the final reconstruction. Each column represents one view, and the size of the column is proportional to the number of particles in that view.

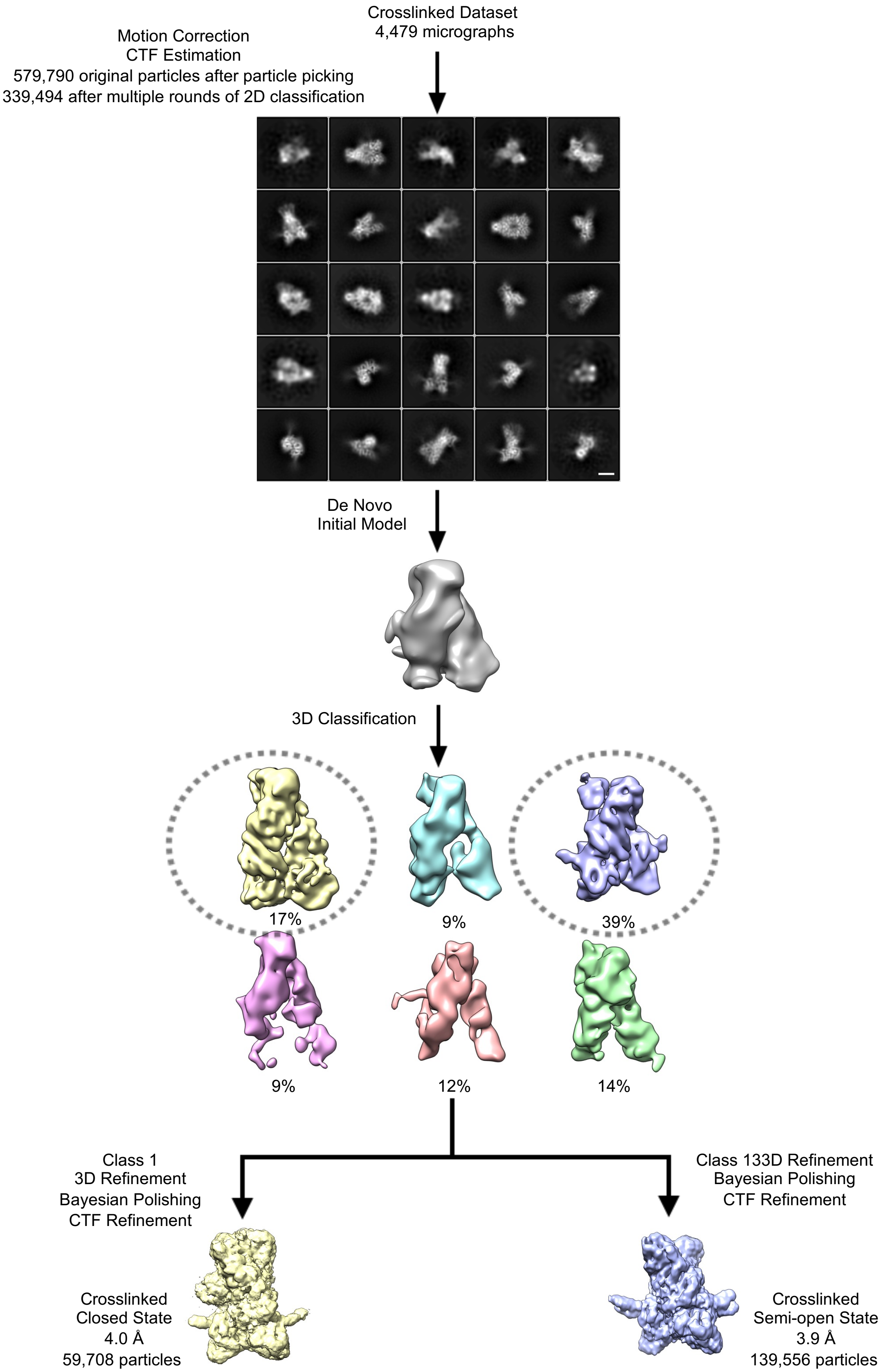

Supplementary Figure 8. Cryo-EM processing workflow for the semi-open state structure solved using cross-linked sample. A schematic representation of the data processing workflow in RELION. The scale bar represents 50 Å. For details, please see the Materials and Methods section of the Image Processing section. Exemplar 2D class averages and cryo-EM maps are depicted at various stages of image processing that yielded both the closed and semi-open states for the cross-linked sample. The number of particles and the global resolution of various classes are indicated.

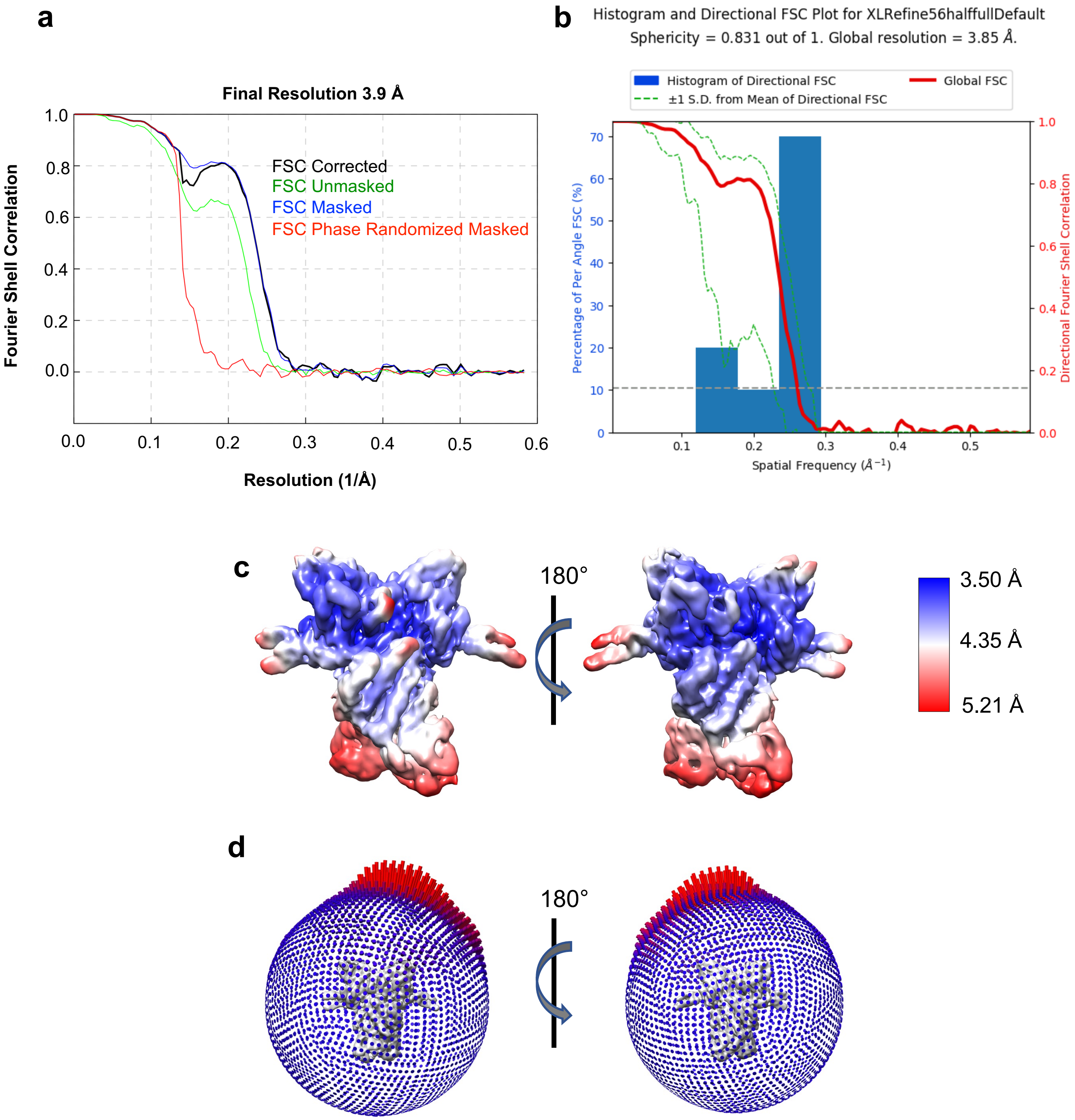

Supplementary Figure 9. Characteristics of the map associated with the HSP90 dimeric complex in the semi-open state obtained using the cross-linked sample. a. The gold-standard FSC curve for the 3D reconstruction of the semi-open state obtained using the cross-linked sample. **b.** Directional FSC histograms and 3DFSC curves are shown in this panel. **c.** Surface representation of the dimeric HSP90 complex obtained using the cross-linked sample, colored to reflect the local resolution calculated by Phenix Local Resolution. **d.** The angular distribution of the final reconstruction. Each column represents one view, and the size of the column is proportional to the number of particles in that view.

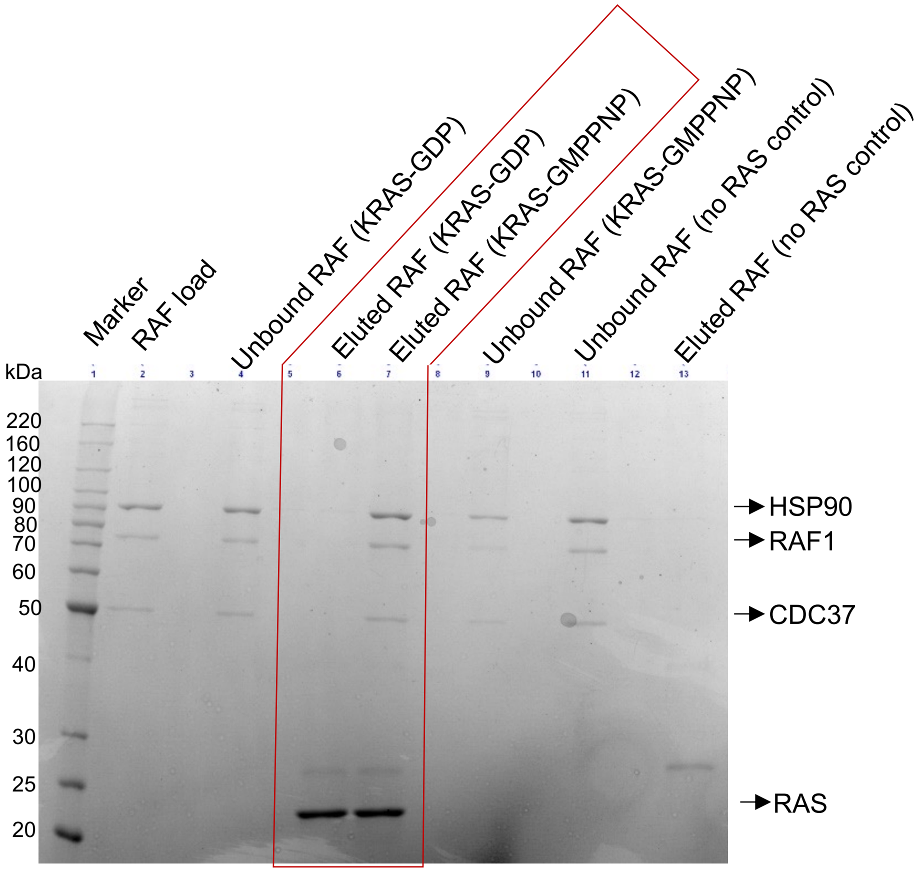

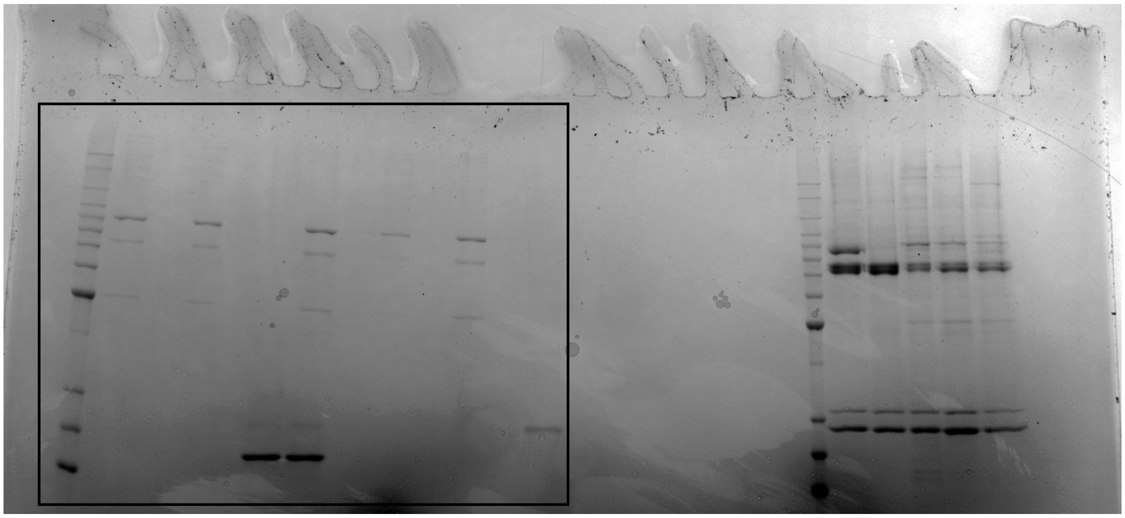

Supplementary Figure 10. Nucleotide-dependent binding of KRAS to RAF1 (RBD-CRD) was studied in the RAF1-HSP90-CDC37 complex. Avi-tagged KRAS (biotinylated) was loaded with either GDP or GMPPNP and immobilized on streptavidin-coated magnetic beads. Subsequently, the closed state complex of RAF1-HSP90-CDC37 was added, and washing and elution steps were conducted to investigate the binding interactions. The results from the pull-down were analyzed using SDS-PAGE, which indicates the presence or absence of each molecular component within the complex. Different lanes on the gel shown in the upper panel represent the specific conditions under which elution and binding interactions were examined. The lower panel shows the uncropped gel, with the boxed part showing the relevant lanes explained in the upper panel.

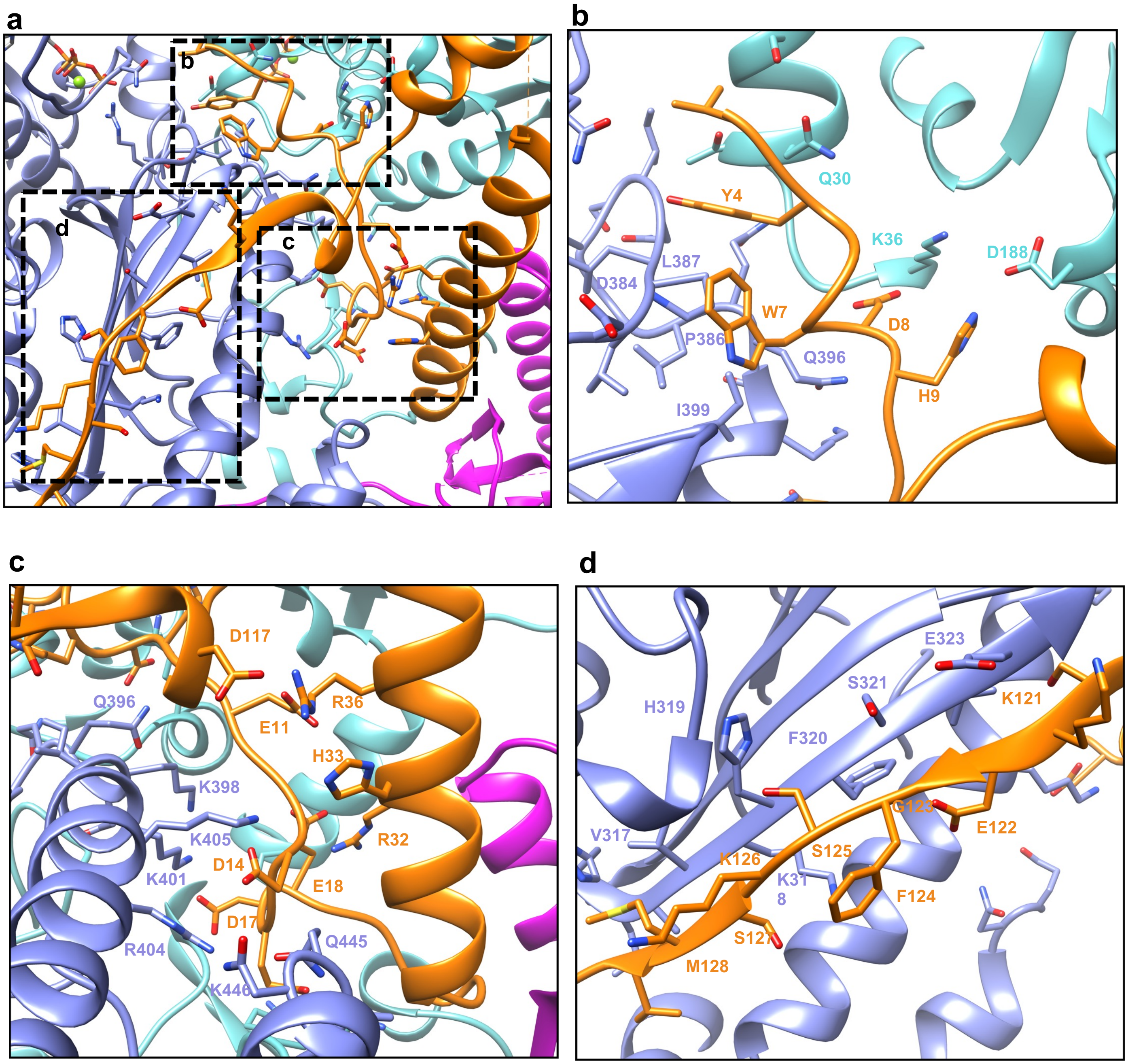

Supplementary Figure 11. Interactions formed by CDC37 with HSP90 protomers in the closed state. **a.** This panel shows the interactions formed by CDC37 residues with the two promoters of HSP90 in the closed state. Three dashed rectangles inside this panel depict three different sets of interactions, which are enlarged in panels b, c, and d. **b.** Interactions formed by the first ten residues of CDC37 with both protomers of HSP90. **c.** Interactions formed by CDC37 residues 10 through 20 with both protomers of HSP90. **d.** Interactions formed by CDC37 residues 121-128, forming a beta-strand with the beta-sheet on HSP90 protomer B.

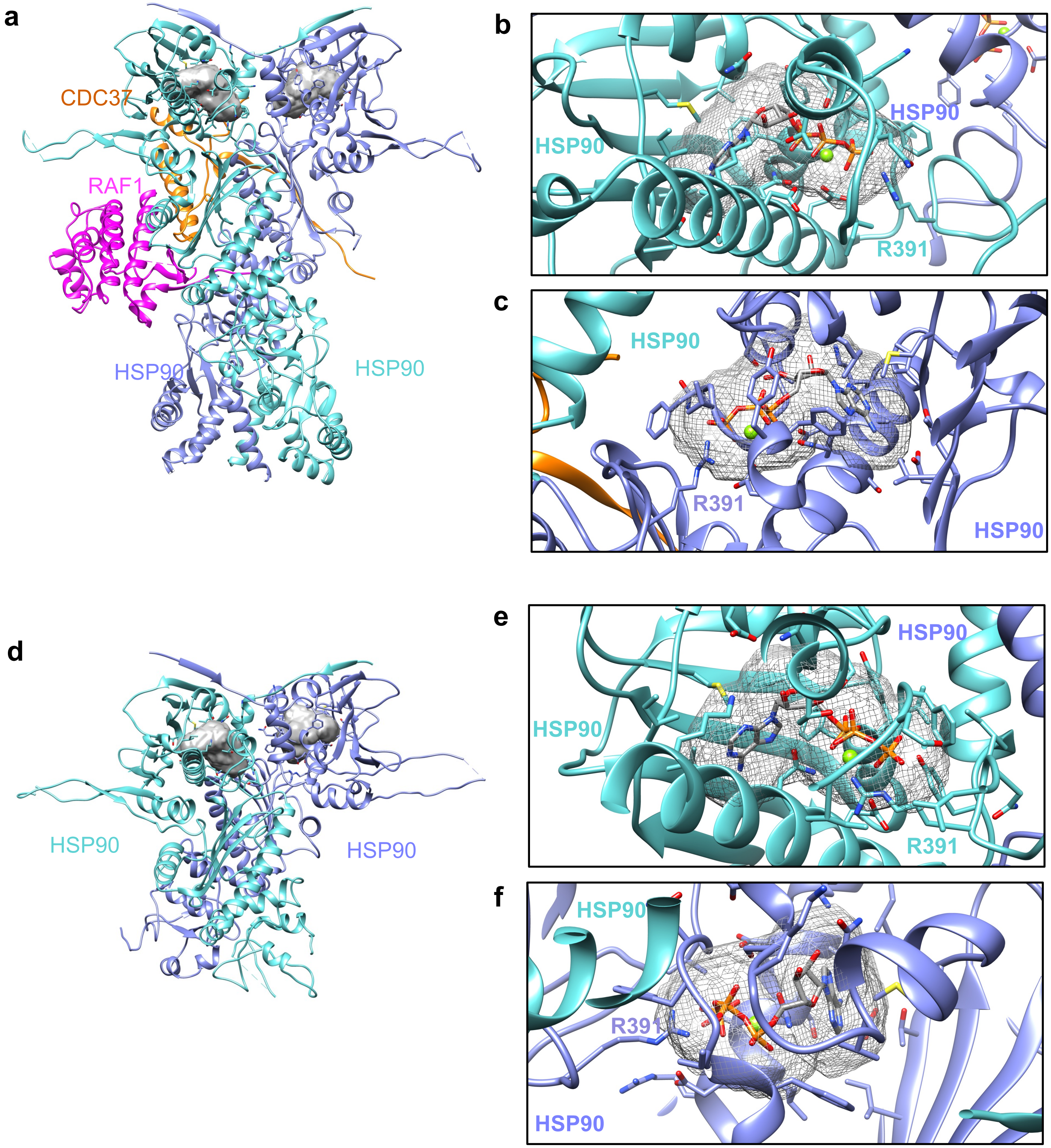

Supplementary Figure 12. Nucleotide-binding site in the N-terminal domain of HSP90 in the closed and semi-open states. a. The overall structure of the closed state complex, showing the electron potential map for the bound nucleotide in the NTD of both protomers of HSP90. **b, c.** An enlarged view of the ATP (ADP + molybdate) binding pocket in (**b**) protomer A and (**c**) protomer B of HSP90, with the electron potential map depicted in gray mesh and the side chain of R391 facing the gamma-phosphate of the ATP. **d.** The overall structure of the semi-open state complex, showing the electron potential map for the bound nucleotide in the NTD of both protomers of HSP90. **e, f.** An enlarged view of the ATP (ADP + molybdate) binding pocket in (**e**) protomer A and (**f**) protomer B of HSP90, with the electron potential map depicted in gray mesh and the side chain of R391 facing the gamma-phosphate of the ATP.

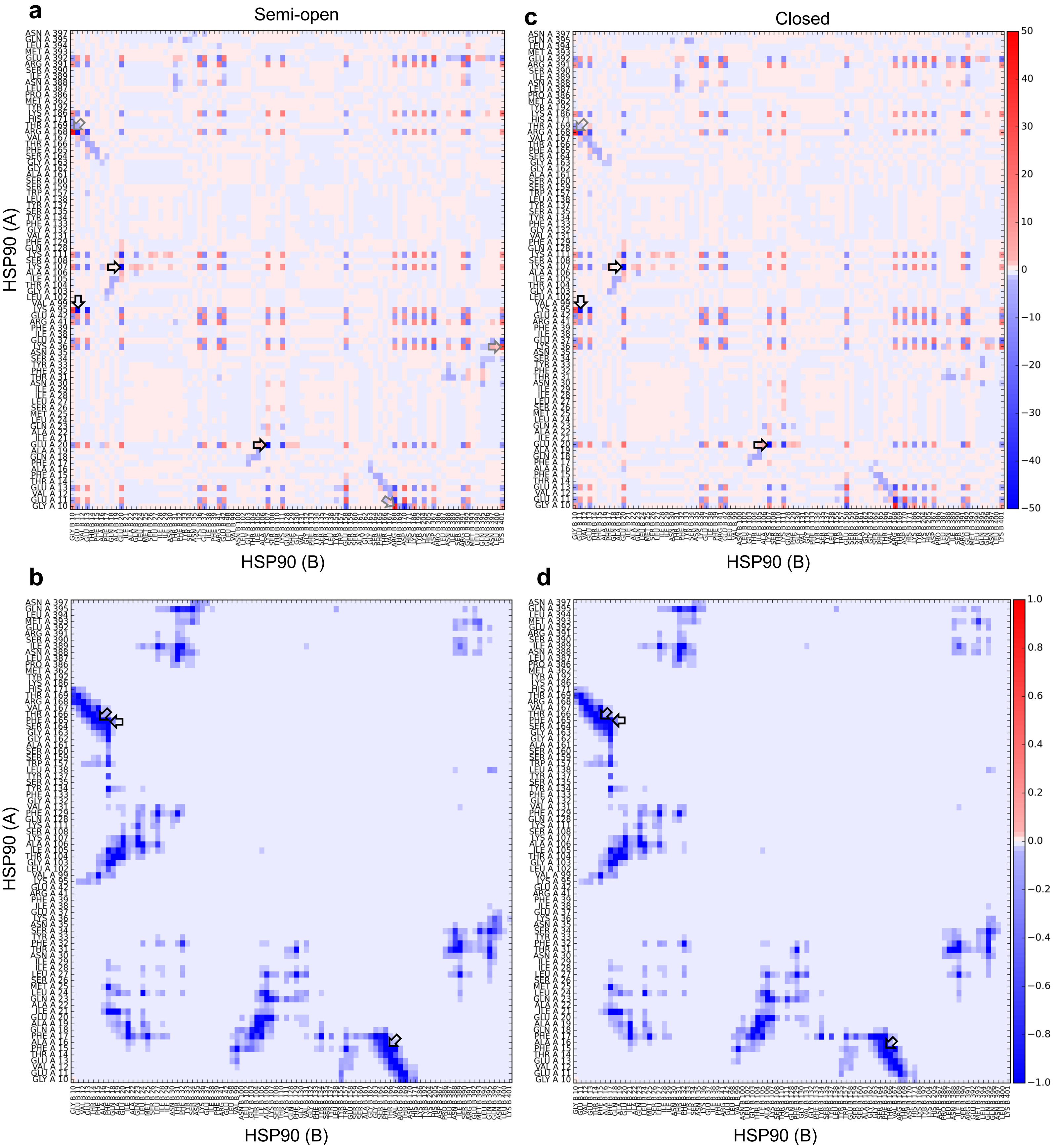

Supplementary Figure 13. Heatmap showing interactions between residues present in the NTD of HSP90 for both semi-open and closed states. Heatmap showing interactions between key residues for semi-open (**a**, **b**) and closed (**c**, **d**) states. Residue interactions were identified within 5 Å proximity to the first 26 residues of the NTD in each protomer (engaging in b-strand swap) for both the semi-open and closed states, and combined into a common selection to facilitate direct comparison between the two states. Panels a and c show the heatmaps for the electrostatic interactions, while panels b and d show van der Waals interactions (favorable in blue, unfavorable in red). The most favorable interactions are indicated by black arrows, whereas other notable interactions are shown using gray arrows.

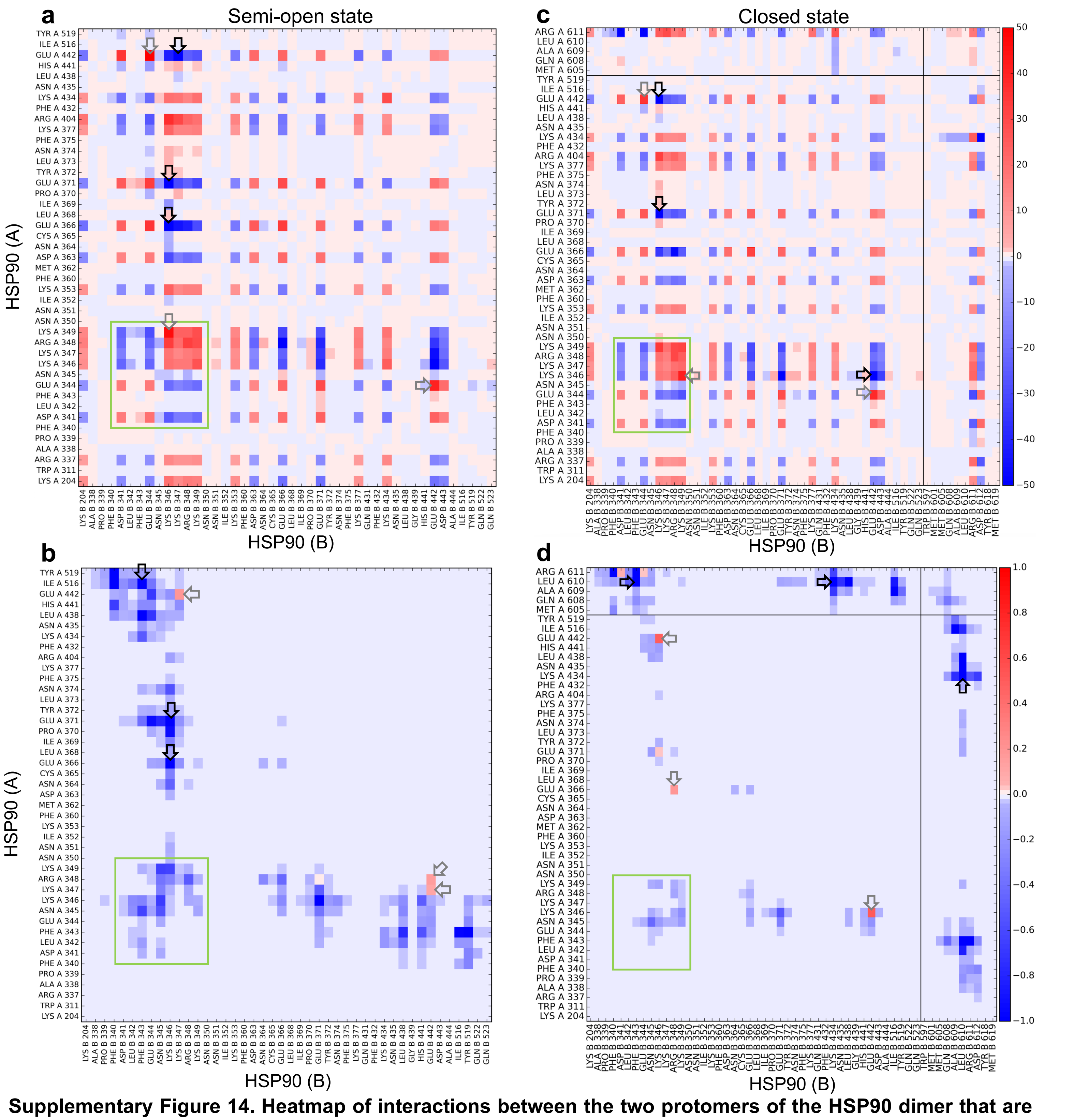

Supplementary Figure 14. Heatmap of interactions between the two protomers of the HSP90 dimer that are proximal to the RAF1 luminal insertion and HSP90 src-loop. Heatmap showing HSP90 interactions for key residues in the semi-open (**a**, **b**) and closed (**c**, **d**) states. Residue interactions were identified within 5 Å of RAF1 luminal residues (418 – 426) and proximal RAF1 residues (427, 428), and within 5 Å of the src-loop (residues 340 – 350) on each protomer of HSP in the closed and semi-open states, and combined into a common selection to facilitate direct comparison between the two states. Both selections were performed on protonated structures. Panels A and C show the heatmaps for electrostatics interactions, while panels B and D show van der Waals interactions (favorable in blue, unfavorable in red). The green box in all four panels highlights the interaction of the src-loops of the two HSP90 protomers with one another. In panels C and D, the top left and bottom right quadrants formed by horizontal and vertical lines show the interactions formed by CTD residues with residues present in NTD and MD, while the top right quadrant shows the CTD residues from two protomers interacting with one another (**panels c**, **d**). The most favorable interactions are indicated by black arrows, whereas other notable interactions are shown using gray arrows.

a

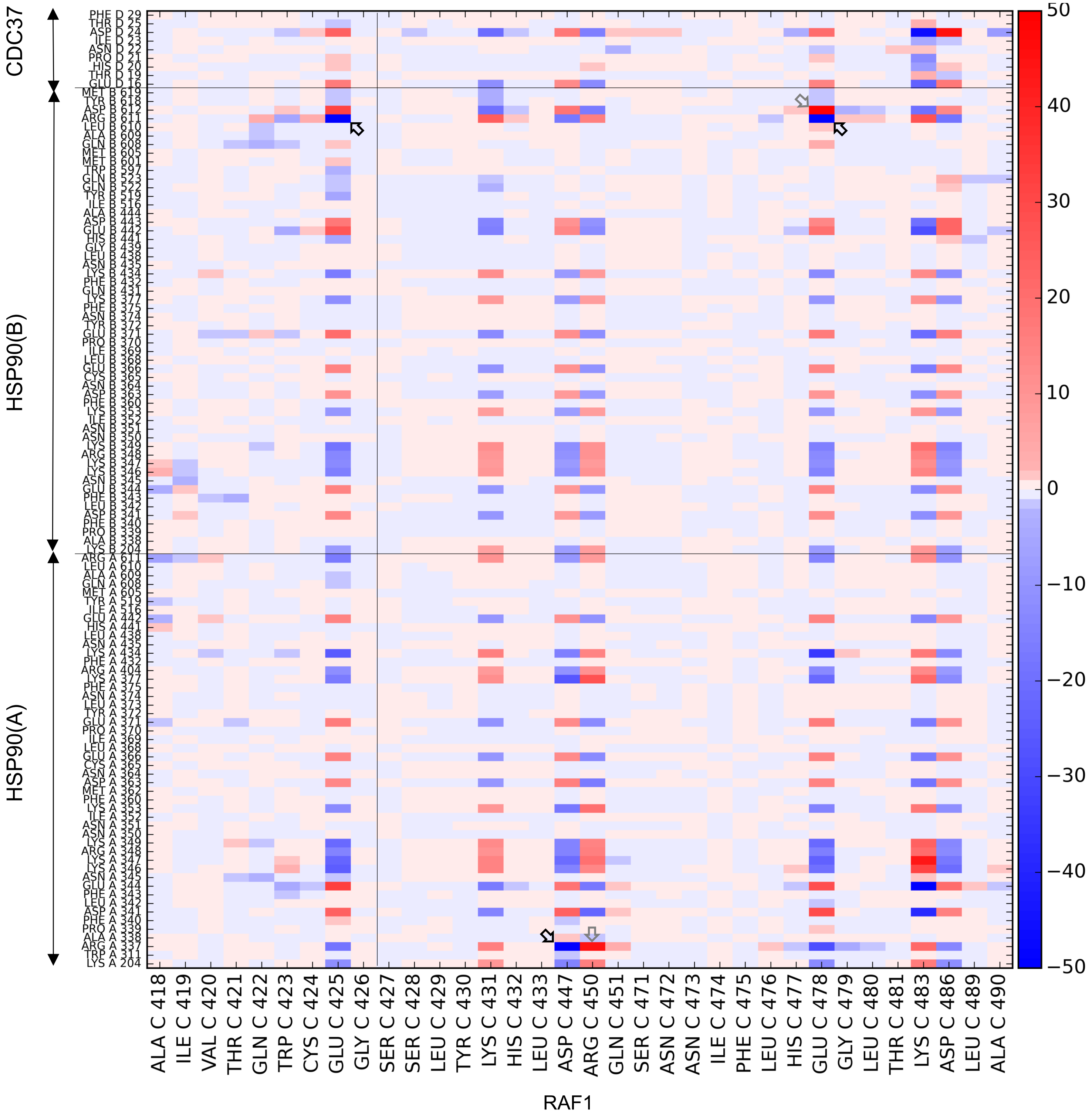
CDC37
HSP90(B)
HSP90(A)
RAF1
ALA C 418
ILE C 419
VAL C 420
THR C 421
GLN C 422
TRP C 423
CYS C 424
GLU C 425
GLY C 426
SER C 427
SER C 428
LEU C 429
TYR C 430
LYS C 431
HIS C 432
LEU C 433
ASP C 447
ARG C 450
GLN C 451
SER C 471
ASN C 472
ASN C 473
ILE C 474
PHE C 475
LEU C 476
HIS C 477
GLU C 478
GLY C 479
LEU C 480
THR C 481
LYS C 483
ASP C 486
LEU C 489
ALA C 490
50
40
30
20
10
0
−10
−20
−30
−40
−50

Continued…

b

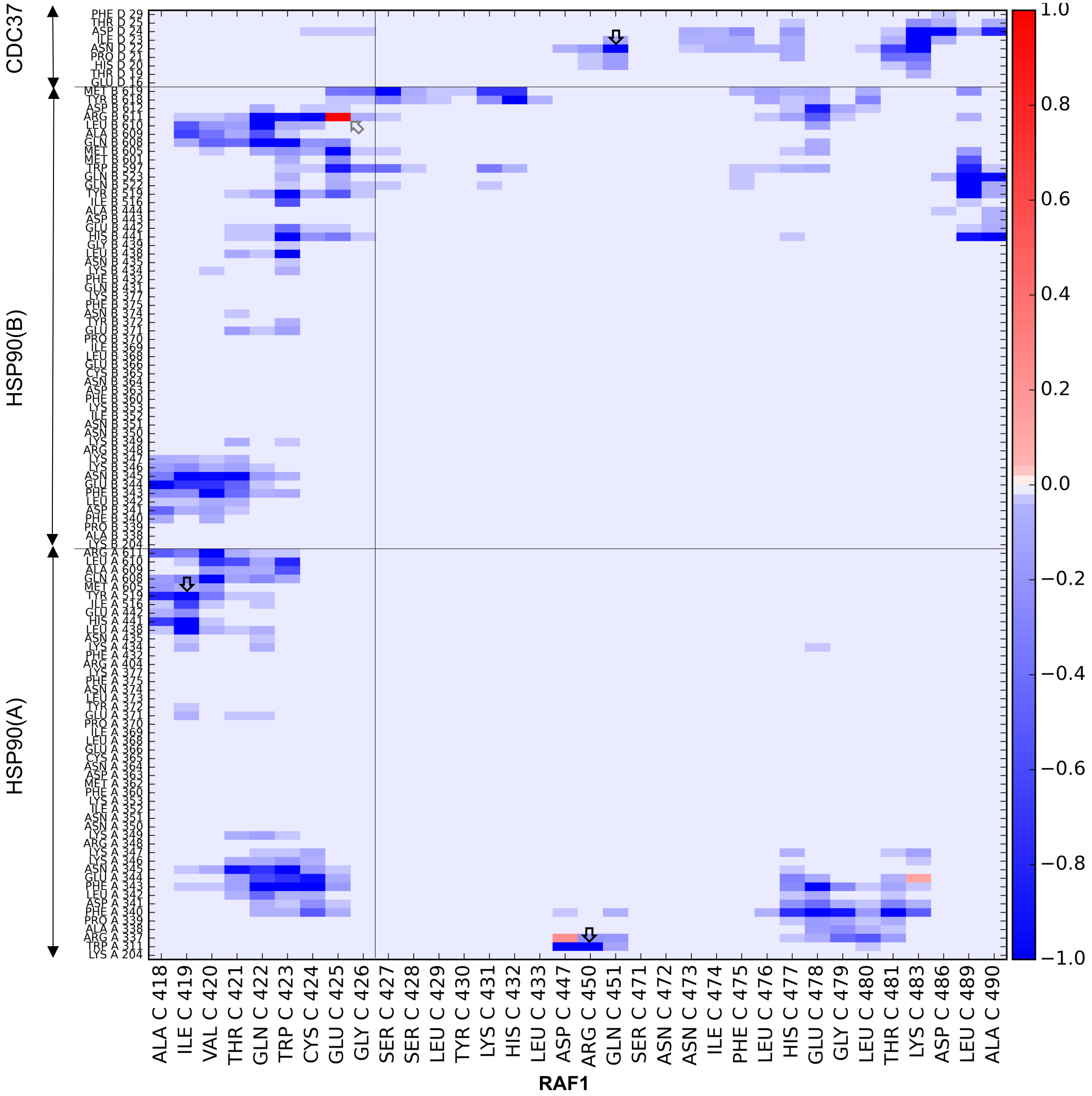

Supplementary Figure 15. Heat map showing interactions of RAF1 with HSP90 and CDC37 in the closed state complex. Panels a and b show the heatmaps for the electrostatics and van der Waals interactions, respectively (favorable in blue, unfavorable in red). Residue interactions were identified within 5 Å of RAF1 luminal residues (418 – 426) and proximal RAF1 residues (427, 428), and within 5 Å of the src-loop (residues 340 – 350) on each protomer of HSP in the closed state, and combined into an overall selection. Both selections were performed on protonated structures. The most favorable interactions are indicated by black arrows, whereas other notable interactions are shown using gray arrows. The luminal residues of RAF1 are denoted to the left of the vertical line.

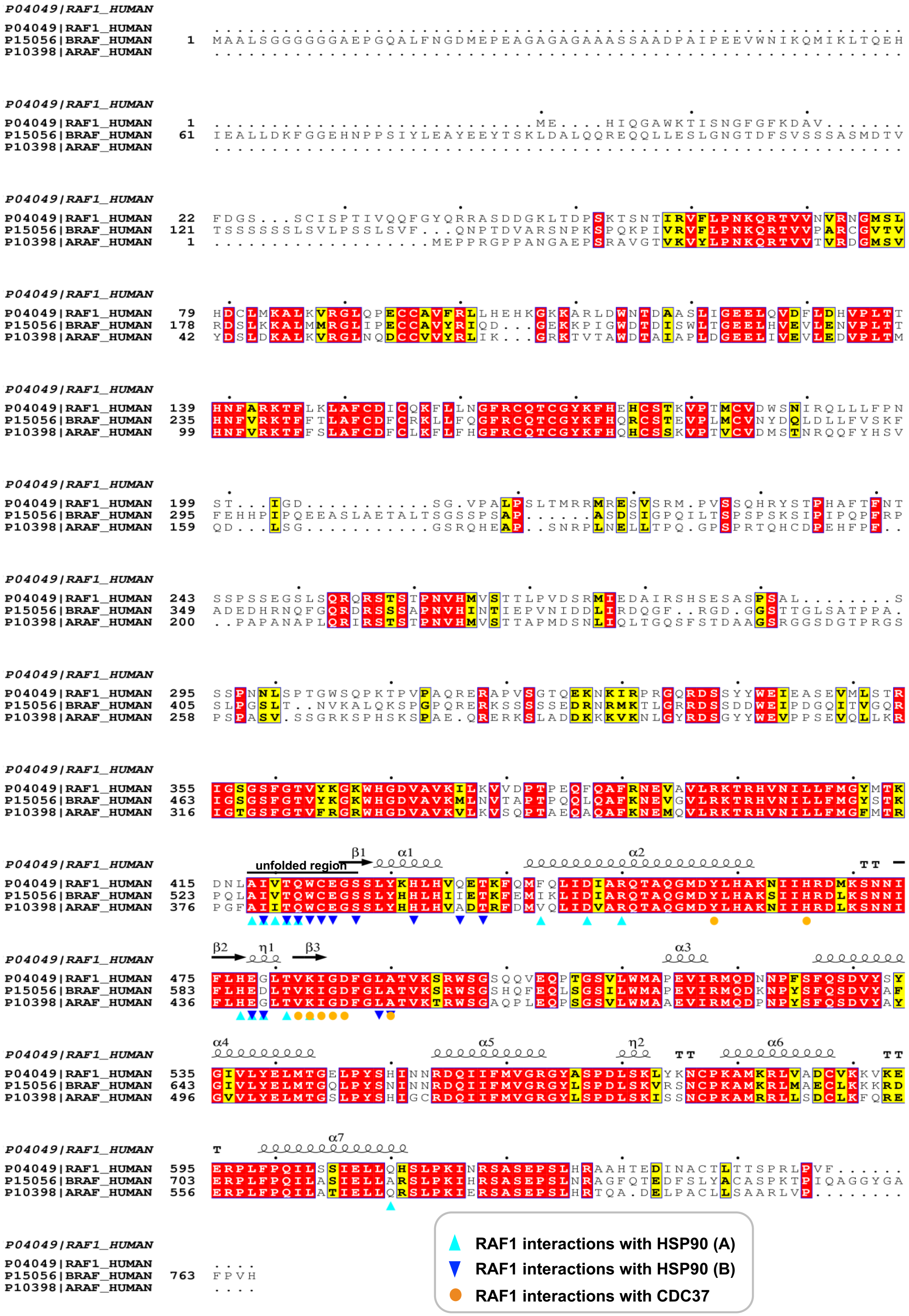

Supplementary Figure 16. Amino acid sequence alignment of Human ARAF, BRAF, and RAF1 (CRAF) proteins. The alignment process was carried out using ClustalW (*Nucleic Acids Res.,* **22**(22), 4673–4680), and the image was generated using ESPript 3.0 (*Nucleic Acids Res.,* **42**(W1), W320-W324). On top of the sequence alignment, the secondary structure of the visible region of the RAF1 in the closed state, along with the unfolded region present in the luminal cavity of the HSP90, are depicted. Furthermore, the interactions formed by RAF1 residues with HSP90 and CDC37 are mapped onto the sequence alignment.

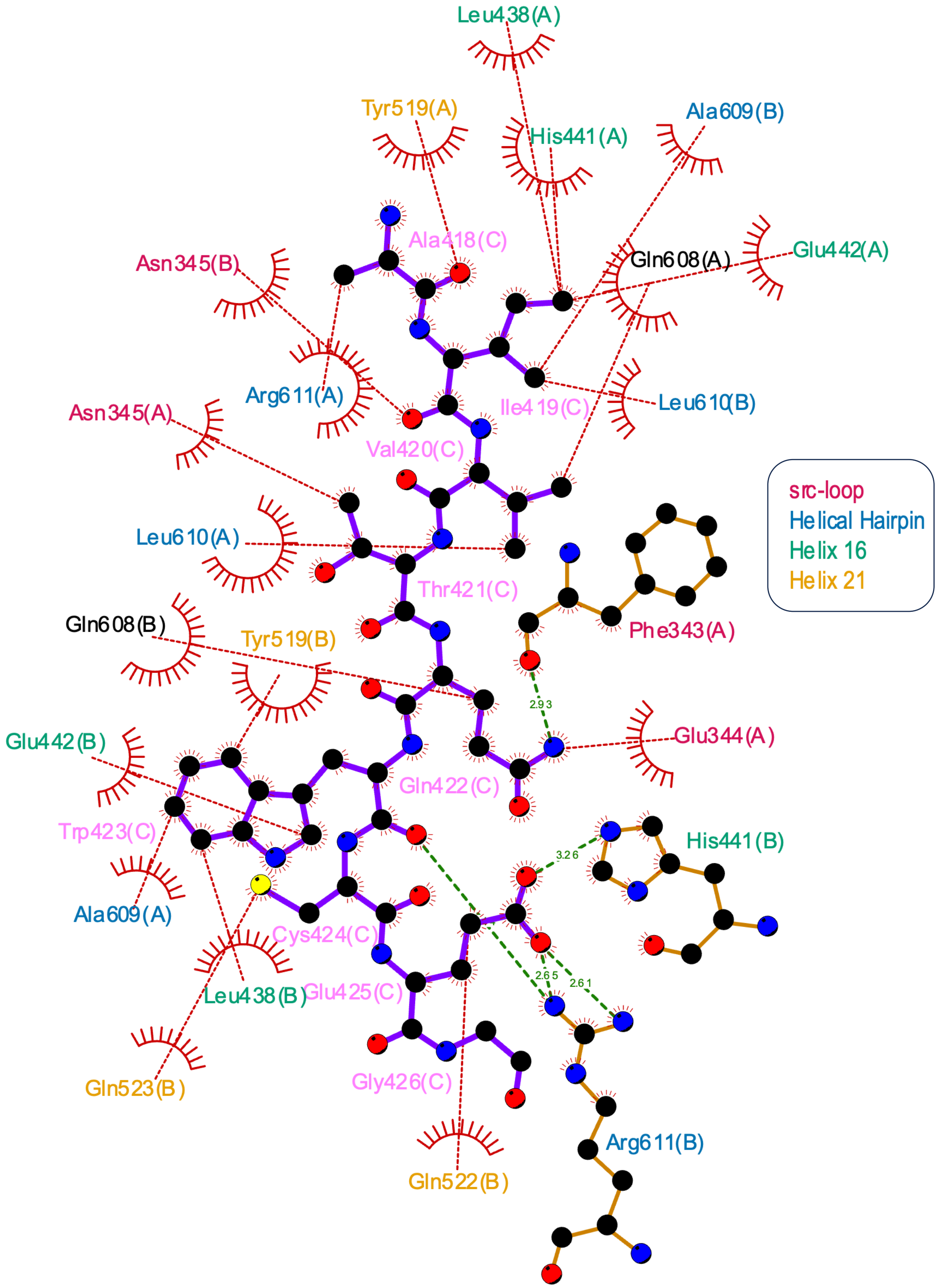

Supplemental Figure 17. Luminal interactions between RAF1 unfolded residues and HSP90. The program LigPlot (*J. Chem. Inf. Model.*, **51**, 2778-2786) was used to generate the interactions. RAF1 unfolded luminal residues are shown in purple. Polar interacting HSP90 residues are shown in tan. HSP90 residues that make van der Waals contacts are shown in red semicircles. Labels indicate the residue name and number, as well as what chain they belong to. Chains A, B, and C correspond to HSP90 protomer A, HSP90 protomer B, and RAF1, respectively. The label colors (red, blue, green, and orange) correspond to the src-loop, Helical Hairpin, Helix 16, and Helix 21, respectively, on either HSP90 protomer, and RAF1 labels are colored magenta. HSP90 residues that are not part of the listed secondary structure elements are labeled in black.

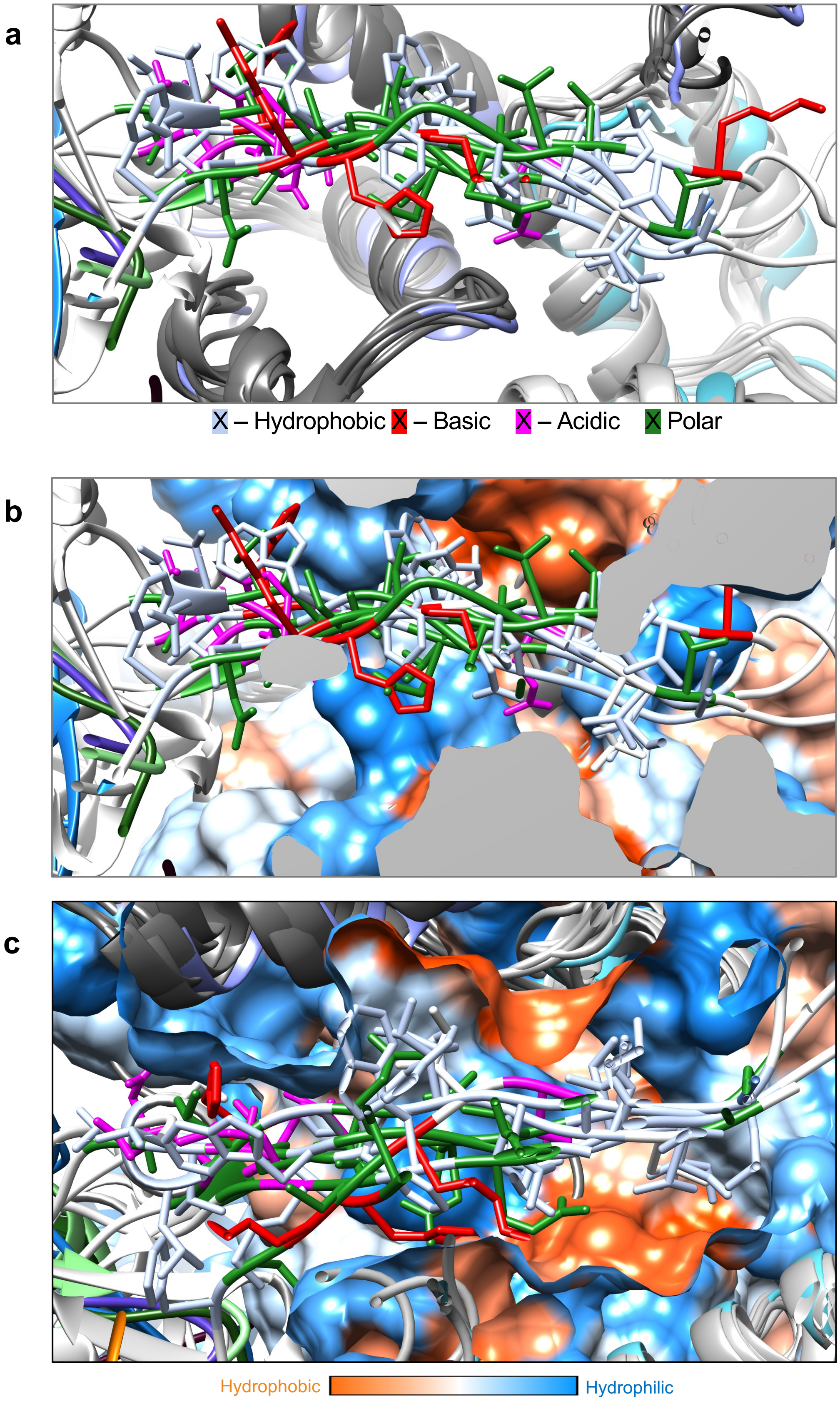

Supplementary Figure 18. Overlay of unfolded client residue interactions within the HSP90 luminal cavity. a. A zoom-in view of the unfolded region of clients in the luminal cavity of HSP90 complexes. The side chains of the clients are shown in a stick model and colored by physiochemical characteristics. **b.** As in panel a, the two HSP90 protomers and CDC37 from our closed state model are shown in surface representation and colored by hydrophobicity. **c.** As in panel b, showing a slightly different view of the luminal cavity.

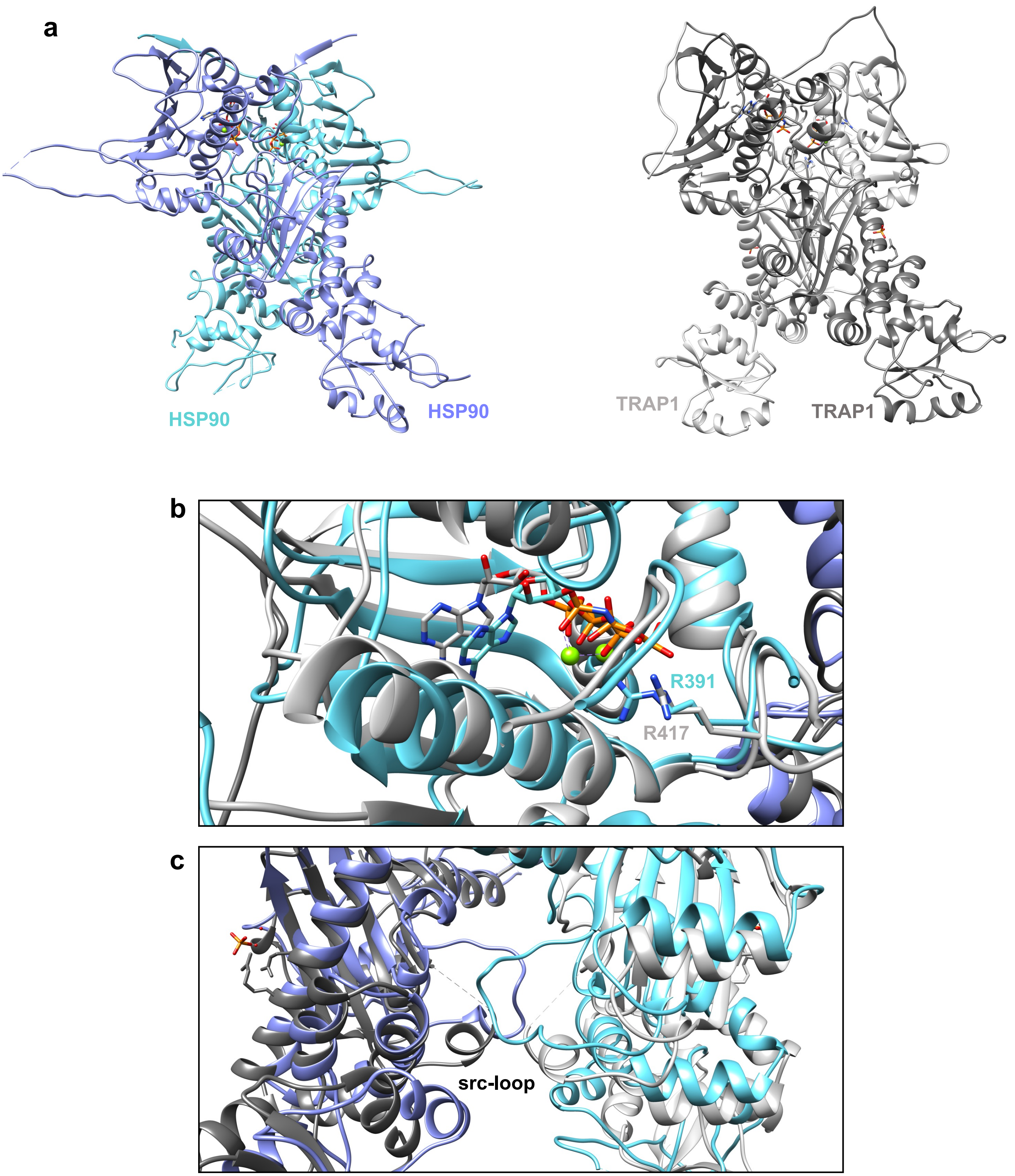

Supplementary Figure 19. Structural comparison of the semi-open state HSP90 dimer with TRAP1 NTD-MD dimer. a. Overall structure of the semi-open HSP90 dimer and crystal structure of TRAP1 (PDB 4IVG) NTD-MD dimer shown in the same orientation. **b.** An enlarged view shows the nucleotide-binding sites in the superposed structures. The semi-open state has ATP, whereas TRAP1 has AMP-PNP modeled in the nucleotide-binding pocket. **c.** A top-down view displays the src-loop region present in the HSP90 semi-open state structure. This region is disordered in the TRAP1 structure.

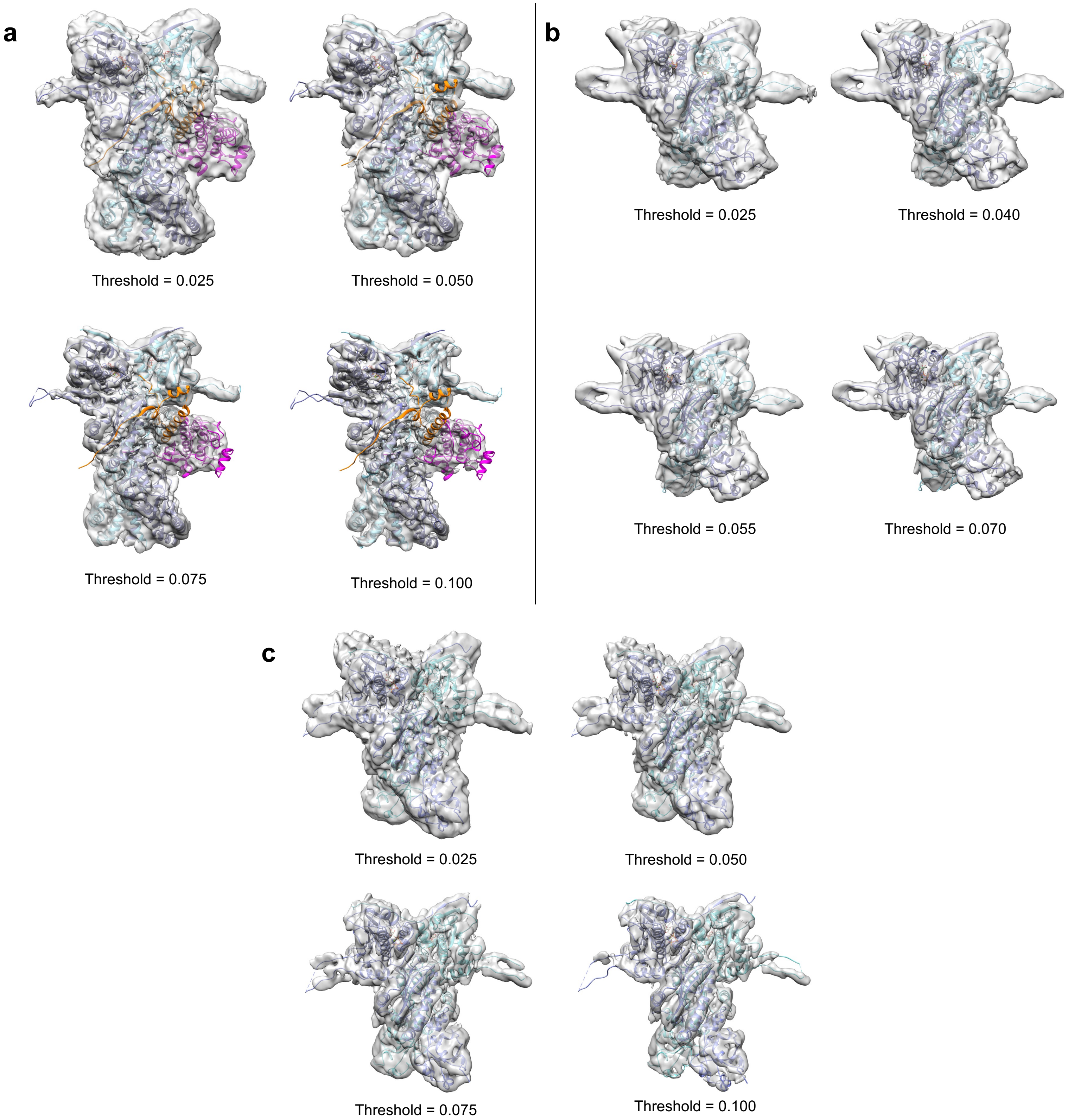

Supplementary Figure 20. Closed state and semi-open state map thresholding. The maps of (**a**) closed, (**b**) semi-open state, and (**c**) cross-linked semi-open are shown at different thresholding cutoffs in combination with a cartoon representation of the underlying proteins in the complexes. The different cutoffs allow for an interpretation of the heterogeneity in electron density in the context of the solved structures.

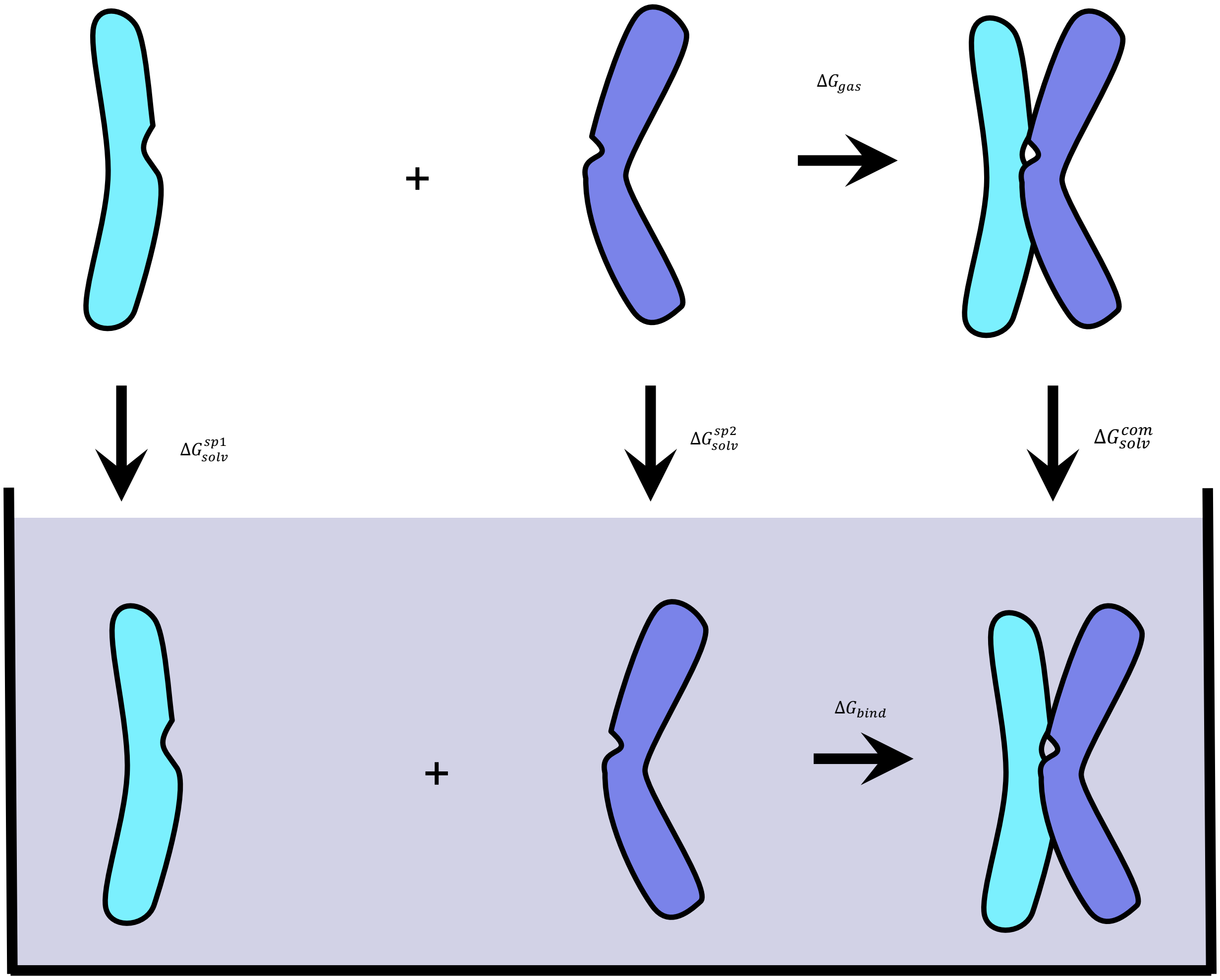

Supplementary Figure 21. Thermodynamic cycle cartoon to illustrate MM-GBSA calculation. Two proteins (colored in blue and cyan) come together to form a complex in vacuum (top), and water (bottom).

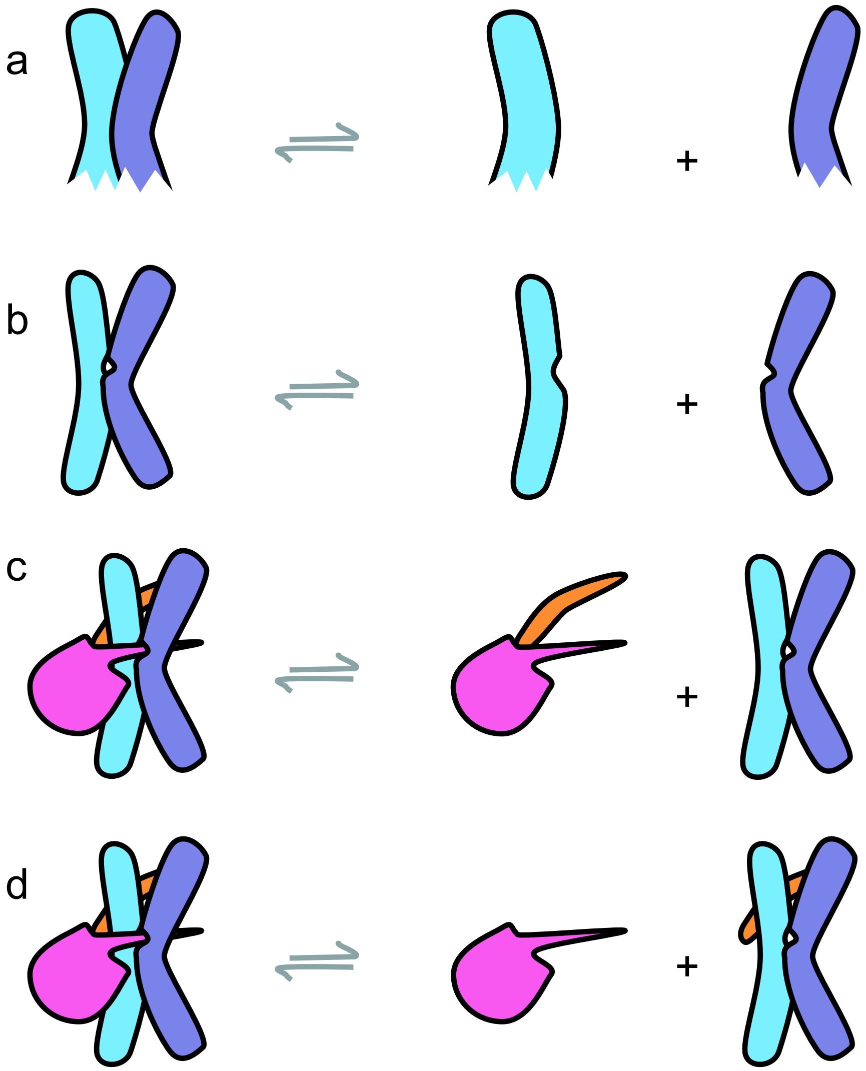

Supplementary Figure 22. Schematic illustration showing estimated binding energies for four different complex formations. HSP90-A, HSP90-B, RAF1, and CDC37 are colored cyan and blue, magenta, and orange, respectively (same color scheme as in Figure 1). Binding energies were calculated by the MM-GBSA method for each replica simulation. **a.** For the semi-open states (both semi-open and semi-open-cross-linked), the binding of the two HSP90 protomers forming the dimer. The jagged edge indicates the missing C-terminal domain. **b.** For the closed state, the binding of HSP90 protomers forming the dimer. **c.** For the closed state, the binding of RAF1 and CDC37 to HSP90 dimer. **d.** For the closed state, the binding of RAF1 to the CDC37-HSP90 dimer complex.

Supplementary Table 1. MM-GBSA analysis to determine the calculated binding free energy between the proteins in closed and semi-open state complexes. Energies for all three complexes are shown for HSP90(A) with HSP90(B): closed, semi-open, and cross-linked semi-open states. Interaction energies between RAF1 and the rest of the complex (HSP90(A), HSP90(B), and CDC37) and interactions between RAF1 and CDC37 with the HSP90 dimer are also shown.

	**Closed state**		**Semi-Open state**		**Semi-Open Cross-linked**	
	Mean	std	mean	std	mean	std
**RAF1— HSP90(A), HSP90(B), CDC37**						
**Replica1**	-148.08	17.43				
**Replica2**	-126.38	17.09				
**Replica3**	-159.22	17.31				
**Replica4**	-128.15	21.99				
**mean**	-140.45					
**stand dev**	15.91					
**stand error**	7.96					
**HSP90(A)—HSP90(B)**						
**Replica1**	-278.29	15.29	-174.22	17.91	-195.05	17.55
**Replica2**	-275.00	17.34	-184.27	15.08	-198.36	18.63
**Replica3**	-280.18	19.94	-189.86	13.95	-201.62	17.05
**Replica4**	-273.40	18.09	-180.86	15.25	-186.94	16.57
**mean**	-276.72		-182.30		-195.49	
**stand dev**	3.08		6.54		6.30	
**stand error**	1.54		3.27		3.15	
**RAF1,CDC37— HSP90(A),HSP90(B)**						
**Replica1**	-251.23	19.54				
**Replica2**	-240.84	21.33				
**Replica3**	-254.12	20.87				
**Replica4**	-221.35	23.17				
**mean**	-241.89					
**stand dev**	14.83					
**stand error**	7.41					
**Energies are in kcal/mol**						

SUPPLEMENTARY METHODS

Supplementary section 1. Introduction.

Here, we supplement the main text with additional details of the computational analysis of the closed and semi-open states, based on a combined 1 μs of atomistic simulation for each of the three complexes. While the literature on computational analyses of HSP90 alone, some in combination with experimental assays, is quite extensive[1,2,3,4,5,6,7], most relevant to the analysis presented here are computational studies conducted on dimeric HSP90 together with CDC37 in complex with a CDK4 client[8,9]. In the work of Stetz and Verkhivker[9], the authors investigated the nature of allosteric interactions of the HSP90 – CDC37 chaperone complex and the CDK4 client and identified putative allosteric client regulation sites using a network analysis formalism. They examined the stability of the complex by performing a computational alanine scan using the FoldX energy function. The authors modeled unresolved loop regions of the complex using the ArchPRED tool. Moreover, they used a specialized molecular simulation method called Discrete Molecular Dynamics, involving a coarse-grained approach in which the protein was represented as a Cα bead model, and in which residue interactions were determined using a discontinuous square well potential. The sampled coarse-grained conformations were subsequently converted and refined into an atomistic representation for further analysis. Using the same HSP90–CDC37–CDK4 client complex, D'Annessa et al.[8] conducted extensive atomistic molecular dynamics simulations of five different nucleotide bound states: Apo, ATP-ATP, ATP-ADP, ADP-ATP, and ADP-ADP, and several short non-equilibrium simulations of the same. Their primary objective was to illustrate the influence of the nucleotide-bound state on the conformational dynamics of the complex, based on an examination of local and long-range differences in residue pair fluctuations within HSP90, the CDC37 chaperone, and the client. Interestingly, they observed that a mixed HSP90 nucleotide-bound state (ATP-ADP or ADP-ATP) resulted in increased conformational fluctuations of the inserted CDK4 luminal residues relative to a doubly ATP-bound state. The authors directly connected two residues that are 55 residues apart (Lys219 and Glu274) due to their structural proximity. The authors also used hydrogen mass repartitioning to increase their simulation timestep to 3 fs. In a separate computational study, Verkhivker utilized coarse-grained sampling with reconstruction, together with a network analysis approach combined with virtual alanine scanning, on the HSP90-HSP70-Hop-GR client complex[10]. Their work shared a similar aim of investigating the effects of allostery on the regulation of interactions of the HSP90 and HSP70 chaperones with GR client. Unlike the previous study of Stetz and Verkhivker on the HSP90-CDC37-CDK4 complex, Verkhivker used a replica-exchange Monte Carlo sampling method in this latter work. The analysis that we present here differs notably from the aforementioned studies: we have utilized standard all-atom molecular dynamics simulations with a conventional forcefield, do not utilize hydrogen mass repartitioning or any other method to enhance molecular sampling, and have not modeled any regions of the HSP90-CDC37-RAF1 complex that were not present in the original EM density. The focus of our analysis is also notably different: in this work, we aim to identify and illustrate important residue pair interactions that differ between the closed HSP90-CDC37-Raf1 and semi-open HSP90 N-terminal and middle domain complexes, utilizing pairwise-residue energetic decomposition to quantify the impact of HSP90 interactions with the client and chaperone relative to the HSP90 semi-open dimeric structure.

Supplementary section 2. Methods.

In the following subsections, we discuss the computational methods deployed in this work. We examined differences in interactions and dynamics between the closed and semi-open states to investigate the consequences of HSP90 interactions with RAF1 and CDC37 (closed state) compared to the HSP90 dimer (semi-open state). We conducted extensive molecular dynamics simulations for both the closed and semi-open states to gain insights into important interactions. Through 1 μs of combined simulations for each state, we identified interacting residue pairs and quantified their strength using per-residue energetic decompositions.

Supplementary section 2.1. Molecular dynamics.

AMBER18[11] and AMBERTools 19[12] or AMBER Tools 20[13] were used. We simulated only what we were able to model in the density and did not build in missing regions. We added capping groups ACE and NME to residues to avoid a positive or negative charge on the termini in areas with a break in the chain and the start of RAF1 in the luminal region. The other chain termini are allowed to be charged. All termini were restrained in the simulation to avoid unphysical movements. The *Build Structures* tool in UCSF Chimera 1.16[14] was used to add nitrogen or carbon to the terminal residue for the NME or ACE capping groups, respectively. Chimera DOCKPrep was used to build in missing sidechains to the protein, and Chimera was used to add hydrogens to the ATP. Antechamber was used to parameterize the ATP by adding AM1-BCC charges[15], and to apply the GAFF2 force field[16]. Antechamber supplemented these parameters with missing definitions, including improper dihedrals (stored in a frcmod file). We performed a multi-step equilibration protocol to prepare all three complexes for molecular dynamics simulations. (See main text **Method** section). During the simulations, the charge of -29 (closed), -25 (semi-open), and -28 (cross-linked semi-open) was neutralized using a uniform neutralizing plasma because counter ions were not used. Only the two structural magnesium ions, which interact with the ATP, were included in all three complexes.

Supplementary section 2.2. RMSF-based B-factor calculation.

We used CPPTRAJ[17] to determine the Root Mean Square Fluctuation (RMSF) by fitting to the starting structure (before equilibration), and using it as the reference for an RMSF calculation. This was done by first reading in all the trajectories through *trajin*, reading in the starting structure as the reference called *startframe*, and then performing '*rms ref [startframe]*' to conduct an RMS fit prior to computation of the RMSF using '*atomicfluct*' with the '*byres bfactor*' flags. The computed B-factor per residue was computed for all states: the closed, semi-open and cross-linked semi-open states, and the maximum B-factor value was identified. All the computed per-residue B-factors from all datasets were then normalized by this value, such that fluctuations of all sets could be directly compared. The normalized B-factor values were then added to the B-factor column of the corresponding closed, semi-open, and cross-linked semi-open state PDB files, and the structures were subsequently viewed in Chimera using the '*rangecolor bfactor*' and associated commands.

Supplementary section 2.3. Principal component analysis (PCA) calculation.

We used CPPTRAJ[17] to compute and visualize the principal components from the combined MD simulation data. This was done through the syntax '*runanalysis modes name Evecs trajout*

[output_trajectory.mdcrd] pcmin -100 pcmax 100 tmode [x]', where *Evecs* corresponds to the eigenvector data file obtained from the combined trajectory data, and *x* corresponds to the desired principal component. Through **Supplementary Movies 1-2**, which show visualizations of the first principal component from the closed and semi-open states, respectively, we get a sense of the relative size of the motion sampled, and the regions of large and small movements.

Supplementary section 2.4. MM-GBSA.

To estimate the binding energies, we calculate the interactions in gas and the solvation terms:

$$\Delta G_{gas} = G_{sys}^{com} - \left(G_{sys}^{sp1} + G_{sys}^{sp2}\right)$$

$$G_{sys} = U + TS$$

$$\Delta G_{gas} = U_{sys}^{com} - \left(U_{sys}^{sp1} + U_{sys}^{sp2}\right) + T\Delta S$$

We neglect the entropic terms in our calculations.

We calculate an approximation to binding energy for two species to form a complex illustrated in the thermodynamic cycle (**Supplementary Figure 21)**. Because we are processing the complex simulation into pieces to calculate the binding energy in gas, all internal energy terms will cancel, and we are left with just the through-space interactions. These can be split into van der Waals (vdW) and electrostatics (Coul) terms:

$$\Delta G_{gas} = \Delta U_{VDW} + \Delta U_{Coul}$$

We define the desolvation term as follows:

$$\Delta\Delta G_{desolv} = \Delta G_{solv}^{com} - \left(\Delta G_{solv}^{sp1} + \Delta G_{solv}^{sp2}\right)$$

This term is the difference between the complex and the two species. This term defines the cost of desolvating the interface of the two species upon binding. The solvation term has two components the GB term (the polar portion) and the apolar term:

$$\Delta G_{solv} = \Delta G_{GB} + \Delta G_{apol}$$

The apolar term is calculated using a linear function of the solvent accessible surface area (SASA).

By looking at the thermodynamic cycle and summing the arrows (**Supplementary Figure 21),** we obtain the following equivalency:

$$\Delta G_{gas} + \Delta G_{solv}^{com} = \Delta G_{solv}^{sp1} + \Delta G_{solv}^{sp2} + \Delta G_{bind}$$

We can rearrange the terms to isolate the bind term:

$$\Delta G_{bind} = \Delta G_{gas} + \Delta G_{solv}^{com} - \left(\Delta G_{solv}^{sp1} + \Delta G_{solv}^{sp2}\right)$$

This gas phase term is modulated by a desolvation term to give us the binding energy:

$$\Delta G_{bind} = \Delta G_{gas} + \Delta\Delta G_{desolv}$$

We break the complex simulation up into pieces. The semi-open state is a two species complex: the HSP90 dimer is made up of two monomers. The closed state is made up of four proteins: two

HSP90 monomers, one RAF1 molecule, and one CDC37 molecule. We constructed four thermodynamic cycles to understand the binding of specific components of the complex and calculated five binding energies (**Supplementary Figure 22** and **Supplementary Table 1**).

Waters were stripped away, and from the four-species-complex simulations, seven trajectories were created and each frame was rescored with Molecular Mechanics-Generalized Born Solvent Accessible Surface Area (MM-GBSA)[18] using the *Sander* executable from AmberTools 19 as follows: We used the Onufriev, Bashford and Case Generalized Born model (igb = 5)[19]. The Solvent Accessible Surface Area is calculated starting with each atom as an icosahedra (gbsa=2). The SASA value is output with no surface tension scaling (surften=1.0). From this SASA value output from *Sander*, we calculate the Apolar energies using an inhouse script as follows: $\Delta G_{apol} = \gamma SASA + \beta$, using the standard constants of $\gamma = 0.00542$ kcal/mol/Å^2 and $\beta = 0.92$ kcal/mol[18,20].

Despite the inaccuracies of the MM-GBSA methods, the method has several strengths that more accurate alchemical methods do not. It is an end-state method which allows us to calculate large changes like protein-protein complex formation, and it allows us to decompose the binding energies into van der Waals, Coulombic, polar solvation and apolar solvation components, which aids us in understanding the molecular driving forces for complex formation. Here we also neglect entropy, which also contributes to the error in our binding prediction. We quantify error within our calculation by running them in quadruplicate and calculating the standard error of the mean among the four replicas. We also look at the variance within each simulation by calculating the standard deviation. These values are reported in **Supplementary Table 1**. The MM-GBSA binding energy may be calculated by running three separate simulations, or one simulation of just the complex. We then break up the complex into pieces. By running one simulation, we reduce error in our calculation, and we can see the MM-GBSA energy as a function of time.

Supplementary section 2.5. Per-residue decomposition.

We calculated a per-residue interaction matrix to understand the important interactions. The molecular mechanics energy function is pairwise additive, and thus we can break up the through-space interactions (vdW and Coulombic) into per-residue components.

We calculate the interaction of every residue of species 1 with every residue of species 2, and store these values in the matrix *U*.

$$\widehat{U}_{ele} = \left[U_{i,j}^{ele}\right] = \left[\sum_{k \in res\ i\ (sp1)} \sum_{l \in res\ j\ (sp2)} U_{ele}(a_k, a_l)\right]$$

The function U_{ele} is used to calculate Coulomb's law for a pair of atoms. a_k and a_l are atoms from species 1 and 2, respectively. The set *res i* and *res j* are residues (you can think of the residues as a set of atoms) from species 1 and 2, respectively. Although we show the equation for the decomposition of electrostatic interactions above, we also compute the vdW decomposition.

This matrix can be displayed as a heatmap. This will show the most strongly interacting pairs of residues. Decompositions are calculated using in-house python scripts (located at *https://github.com/tbalius/teb_scripts_programs/tree/master/py_amber_reader*).

Supplementary section 2.6. HSP90 Luminal Cavity Volume Calculation.
The luminal cavity volume was calculated as follows. The program *DMS*[21] (https://www.cgl.ucsf.edu/chimera/docs/UsersGuide/midas/dms1.html) was used to calculate the molecular surface. Furthermore, because of the size of the system, the molecular surface was only calculated for a subset of residues. The program *filt* distributed with *UCSF DOCK 3.7* was used to find the residues close to the inserted RAF region to select the residues included in the surface calculation. The program *sphgen*[22] provided with *UCSF DOCK 3.7* was used to calculate the inverse image of the molecular surface. Sphgen does this by growing a sphere at each surface point along the orthonormal vector until another surface point is encountered. The value of 0.0 is used for the halt condition of how close the sphere can be to another surface point (this condition may be negative if some overlap can be tolerated with the surface). The program *sphgen* clusters the spheres to define pockets, but also writes out all the spheres that it generated as Cluster 0, which is what we use. The program *read_write_sph_mod.py*, which we make available in *teb_scripts_programs* (*https://github.com/tbalius/teb_scripts_programs/tree/master/zzz.scripts*), is used to remove spheres with radii that are bigger than 4.0 angstroms. We used the program *selected_spheres.sph*, distributed with *UCSF DOCK6.9*, to keep only the spheres within 5.5 angstroms of the luminal inserted region of RAF (we tried different distance values and visually inspected them to arrive at this distance, which we determined filled up the cavity and does not go outside). Although we did not need to, spheres can be culled by hand to ensure that the volume is calculated on just the cavity of interest. We then used the *volume_cal_sph.py* (located in *https://github.com/tbalius/teb_scripts_programs/tree/master/zzz.scripts*) to calculate the volume taken up by the spheres. The program does this by bounding the spheres with a box and filling the box with a grid. We use a grid resolution of 0.2 angstroms (the finer the resolution, the more accurate the volume calculation). We calculate the volume of the box as follows:

$$V_B = width \times height \times depth$$

We determine the number of grid points that are inside the spheres, N_C, and the number of grid points in the box, N_B. The volume of the cavity is defined as follows:

$$V_C = V_B \frac{N_C}{N_B}$$

The *volume_cal_sph.py* also outputs a grid file in dx format, where the points inside the spheres are indicated with a value of one, and those outside with a value of zero, which allows us to visualize the calculated volume. We used this grid to make **Figure 3e**.

The molecular volume of the RAF unfolded region (that which is inserted into the luminal cavity) is calculated by converting each atom into a sphere with the atomic radius set to the van der Waals radii used in DOCK3.7. We then calculate the volume as described above.

Supplementary section 3. Caveats.

A few caveats from our simulations warrant airing. **1. Restraints.** As mentioned in **Supplementary Methods,** we added restraints to the ends of missing regions and capped these regions. This is common practice, but can result in artifacts, including inhibiting movements in the restrained regions which might be unphysical. However, leaving these regions unrestrained would result in more issues**. 2. Missing regions.** Large regions are missing from the model. We opted to leave these regions unmodeled. However, we believe that such an approach is warranted, given that we are unable to confidently model in these regions, and posit that it is better to model what we know definitively instead of speculating on a structural region that we do not have reliable experimental data for. **3. Convergence.** The simulations all seem to be sampling different regions of conformational space (evidenced by looking at the first two principal components of the combined trajectories). It is difficult to judge how long is long enough. To address convergence, we generated time series plots of the system's potential, kinetic and total energy, as well as the MM-GBSA energies. In addition to visually looking at these plots, we calculated Block Average Standard Error of the Mean, Autocorrelation functions, and the slope of the trend line. We also plotted RMSD time series. The data seem reasonably well behaved, these give us a sense of the internal error, and correlated trends. We see that the energies go up and down and seem to fluctuate about a mean value. The analysis also helped convince us that our equilibration procedure is reasonable. We ran four 250ns trajectories calculated MM-GBSA binding values and presented a statistical analysis in **Supplemental Table 1** quantifying the variance within each trajectory and among the trajectories. Reassuringly, despite sampling different portions of conformational space, the binding energies are reasonably close in value giving us confidence in the mean value (within the standard error). **4. Resolution**. Because the models were obtained from a 3.9, 3.7 and 3.5 Å resolution, there is the possibility that other models could also conceivably fit the density. However, we have constructed the most reasonable model we could and used this to obtain atomic models of the three complexes. This gave us a reasonable starting point for our simulations. We also performed simulations from earlier models and obtained similar results, although sampling in the key luminal region differed somewhat.

These caveats do not alter the findings realized from the simulations. To support the structural work, our simulations have been used to estimate binding energies of complex formation, and to quantify the strength of residue-residue interactions among the proteins. Through the residue-residue energetic analysis, we can speak to interactions (including estimated strengths) that we would not otherwise have confidence in based on our 3.5-3.9 Å structures alone. Furthermore, our computationally determined root mean square fluctuation (RMSF) visualizations for each solved structure can be directly related to the experimental structure factors, and the interaction energy values that we calculate through the residue-residue potential energy decomposition, and the observations that we draw from the dynamics of the system provide a framework for further mutagenesis experiments and binding assays. In this sense, our molecular simulation and quantitative analysis provides a means to further augment and interpret the structural data that has been obtained through cryo-EM.

Supplementary section 4. Computational Summary.

By comparing the closed and semi-open states using energetic decomposition analysis, we can observe the stabilizing contribution of the CTD (HSP90) towards the formation of the closed-state complex, as well as the contribution of the NTD (HSP90) in stabilizing the HSP90 dimer in both states. Moreover, our comparative analysis reveals a redistribution of vdW contacts, originally present between the MD residues of HSP90 and luminal and peripheral residues of RAF1 in the closed state, to contacts made between the MD residues of HSP90 in the semi-open state. Through PCA, we were further able to quantify and observe the motions contributing to the variance in our molecular simulation data, noting that the largest uncorrelated motion reveals asymmetric fluctuations in the HSP90 protomers in both the closed and semi-open states. Our simulations support and augment the cryo-EM structural work by providing quantitative analysis of system component interaction energetics through MM-GBSA and pairwise-residue energetic decomposition. The interaction energy values that we provide, and the observations that we draw from the dynamics of the system provide a framework for further mutagenesis experiments and binding assays. Furthermore, our computationally determined RMSF visualizations (**Figs. 1e**, **6f**, **6g**) can be directly related to the structure factors for each of the solved structures.

Supplementary References

1. Colombo G, Morra G, Meli M, Verkhivker G. Understanding ligand-based modulation of the Hsp90 molecular chaperone dynamics at atomic resolution. *Proceedings of the National Academy of Sciences* **105**, 7976-7981 (2008).

2. Dixit A, Verkhivker GM. Probing molecular mechanisms of the Hsp90 chaperone: biophysical modeling identifies key regulators of functional dynamics. *PLoS One* **7**, e37605 (2012).

3. Jussupow A, Lopez A, Baumgart M, Mader SL, Sattler M, Kaila VRI. Extended conformational states dominate the Hsp90 chaperone dynamics. *J Biol Chem* **298**, 102101 (2022).

4. Kandzia F, Ostermeir K, Zacharias M. Global Dynamics of Yeast Hsp90 Middle and C-Terminal Dimer Studied by Advanced Sampling Simulations. *Front Mol Biosci* **6**, 93 (2019).

5. Luo A*, et al.* Identification of AtHsp90.6 involved in early embryogenesis and its structure prediction by molecular dynamics simulations. *R Soc Open Sci* **6**, 190219 (2019).

6. Morra G, Verkhivker G, Colombo G. Modeling signal propagation mechanisms and ligand-based conformational dynamics of the Hsp90 molecular chaperone full-length dimer. *PLoS Comput Biol* **5**, e1000323 (2009).

7. Rehn A*, et al.* Allosteric Regulation Points Control the Conformational Dynamics of the Molecular Chaperone Hsp90. *J Mol Biol* **428**, 4559-4571 (2016).

8. D'Annessa I, Moroni E, Colombo G. Visualizing the Dynamics of a Protein Folding Machinery: The Mechanism of Asymmetric ATP Processing in Hsp90 and its Implications for Client Remodelling. *J Mol Biol* **433**, 166728 (2021).

9. Stetz G, Verkhivker GM. Functional Role and Hierarchy of the Intermolecular Interactions in Binding of Protein Kinase Clients to the Hsp90-Cdc37 Chaperone: Structure-Based Network Modeling of Allosteric Regulation. *J Chem Inf Model* **58**, 405-421 (2018).

10. Verkhivker GM. Exploring Mechanisms of Allosteric Regulation and Communication Switching in the Multiprotein Regulatory Complexes of the Hsp90 Chaperone with Cochaperones and Client Proteins: Atomistic Insights from Integrative Biophysical Modeling and Network Analysis of Conformational Landscapes. *J Mol Biol* **434**, 167506 (2022).

11. Case DA*, et al.* AMBER 2018, University of California, San Francisco.) (2018).

12. Case DA*, et al.* AMBERTools 2019, University of California, San Francisco.) (2019).

13. Case DA*, et al.* AMBERTools 2020, University of California, San Francisco.) (2020).

14. Pettersen EF*, et al.* UCSF Chimera--a visualization system for exploratory research and analysis. *J Comput Chem* **25**, 1605-1612 (2004).

15. Jakalian A, Jack DB, Bayly CI. Fast, efficient generation of high-quality atomic charges. AM1-BCC model: II. Parameterization and validation. *Journal of Computational Chemistry* **23**, 1623-1641 (2002).

16. He X, Man VH, Yang W, Lee T-S, Wang J. A fast and high-quality charge model for the next generation general AMBER force field. *The Journal of Chemical Physics* **153**, 114502 (2020).

17. Roe DR, Cheatham TE, III. PTRAJ and CPPTRAJ: Software for Processing and Analysis of Molecular Dynamics Trajectory Data. *Journal of Chemical Theory and Computation* **9**, 3084-3095 (2013).

18. Massova I, Kollman PA. Combined molecular mechanical and continuum solvent approach (MM-PBSA/GBSA) to predict ligand binding. *Perspectives in Drug Discovery and Design* **18**, 113-135 (2000).

19. Onufriev A, Bashford D, Case DA. Exploring protein native states and large-scale conformational changes with a modified generalized born model. *Proteins: Structure, Function, and Bioinformatics* **55**, 383-394 (2004).

20. Sitkoff D, Sharp KA, Honig B. Accurate Calculation of Hydration Free Energies Using Macroscopic Solvent Models. *The Journal of Physical Chemistry* **98**, 1978-1988 (1994).

21. Richards FM. Areas, volumes, packing and protein structure. *Annu Rev Biophys Bioeng* **6**, 151-176 (1977).

22. Kuntz ID, Blaney JM, Oatley SJ, Langridge R, Ferrin TE. A geometric approach to macromolecule-ligand interactions. *J Mol Biol* **161**, 269-288 (1982).
