# Supplementary material for: Structural dynamics of RAF1-HSP90-CDC37 and HSP90 complexes reveal asymmetric client interactions and key structural elements
Source: Commun Biol. 2024 Mar 2;7:260. doi: 10.1038/s42003-024-05959-3 (PMC10908828; doi:10.1038/s42003-024-05959-3)
Supplement: Supplementary file 3 — Description of Additional Supplementary Files [file 42003_2024_5959_MOESM3_ESM.pdf]

Description of Additional Supplementary Files

File name: Supplementary Movie 1
Description: The motion occurring along the first principal component, as obtained from 1 ms of combined molecular simulation, for the closed state. The structure is shown rotating about the y-axis, while the component motion rocks forward and backward.

File name: Supplementary Movie 2
Description: The motion occurring along the first principal component, as obtained from 1 ms of combined molecular simulation, for the semi-open state. The structure is shown rotating about the y-axis, while the component motion rocks forward and backward.

File name: Supplementary Movie 3
Description: 3D Variability Analysis (3DVA) of the closed state structure, obtained from cryoSPARC.

File name: Supplementary Movie 4
Description: 3D Variability Analysis (3DVA) of the semi-open state structure, obtained from cryoSPARC.
